# Evaluation of new alternative methods for the identification of estrogenic, androgenic and steroidogenic effects: a comparative in vitro*/*in silico study

**DOI:** 10.1007/s00204-023-03616-y

**Published:** 2023-10-11

**Authors:** A. Najjar, A. Wilm, J. Meinhardt, N. Mueller, M. Boettcher, J. Ebmeyer, A. Schepky, D. Lange

**Affiliations:** Beiersdorf AG, Beiersdorfstr. 1-9, 20245 Hamburg, Germany

**Keywords:** Endocrine-disrupting potential, NAMs, EATS, Oestrogen, Androgen, Steroidogenesis, In vitro/In silico, NGRA, ToxCast

## Abstract

A suite of in vitro assays and in silico models were evaluated to identify which best detected the endocrine-disrupting (ED) potential of 10 test chemicals according to their estrogenic, androgenic and steroidogenic (EAS) potential compared to the outcomes from ToxCast. In vitro methods included receptor-binding, CALUX transactivation, H295R steroidogenesis, aromatase activity inhibition and the Yeast oestrogen (YES) and Yeast androgen screen (YAS) assays. The impact of metabolism was also evaluated. The YES/YAS assays exhibited a high sensitivity for ER effects and, despite some challenges in predicting AR effects, is a good initial screening assay. Results from receptor-binding and CALUX assays generally correlated and were in accordance with classifications based on ToxCast assays. ER agonism and AR antagonism of benzyl butyl phthalate were abolished when CALUX assays included liver S9. In silico final calls were mostly in agreement with the in vitro assays, and predicted ER and AR effects well. The efficiency of the in silico models (reflecting applicability domains or inconclusive results) was 43–100%. The percentage of correct calls for ER (50–100%), AR (57–100%) and aromatase (33–100%) effects when compared to the final ToxCast call covered a wide range from highly reliable to less reliable models. In conclusion, Danish (Q)SAR, Opera, ADMET Lab LBD and ProToxII models demonstrated the best overall performance for ER and AR effects. These can be combined with the YES/YAS assays in an initial screen of chemicals in the early tiers of an NGRA to inform on the MoA and the design of mechanistic in vitro assays used later in the assessment. Inhibition of aromatase was best predicted by the Vega, AdmetLab and ProToxII models. Other mechanisms and exposure should be considered when making a conclusion with respect to ED effects.

**Supplementary Information:**

The online version contains supplementary material available at 10.1007/s00204-023-03616-y.

## Introduction

An evaluation of the potential of a chemical to cause endocrine disruption (ED) is an important aspect of a safety evaluation since the endocrine system is responsible for the production and regulation of hormones, which are crucial for the normal development, growth and functioning of the body. Endocrine-disrupting chemicals can interfere with normal hormone signalling, leading to a wide range of adverse health effects, including reproductive and developmental abnormalities, cancer, obesity and neurological disorders (WHO 2012). Oestrogen (ER) agonists or antagonists can affect processes, such as reproduction and development, whilst chemicals that bind to the androgen receptor (AR) can affect sexual development and function (Amir et al. 2021). The evaluation of ED is challenging since no single assay can predict all pathways involved. Therefore, a combination of assays is necessary for a comprehensive assessment of ED potential. As a result, regulatory agencies, such as the OECD (OECD 2018), European Food Safety Authority (EFSA), the European Chemicals Agency (ECHA) and the Joint Research Centre (JRC) (ECHA et al. 2018), have developed guidelines and testing strategies to evaluate the ED potential of chemicals. These guidelines typically include a range of in vitro and in vivo assays to assess the potential effects of chemicals on hormone signalling pathways, as well as their toxicity and exposure potential. This mechanistic approach is also in accordance with the next-generation risk assessment (NGRA) approach described by others for the assessment of cosmetic ingredients e.g. parabens (Alexander-White et al. 2022). In an NGRA, the assessment is conducted in a tiered fashion, starting with an assessment of the structural and physicochemical properties, as well as in silico information to inform on a mode of action (MoA) and form a hypothesis which is then investigated using targeted testing (using in vitro bioassays) and linking these with blood and tissue concentrations.

The aim of this study was therefore to identify which in silico models best predicted ER, AR and aromatase inhibition and whether these could be combined with the YES/YAS assays in an initial screen of chemicals, which could be used in the early tiers of an NGRA to inform on the MoA and the design of mechanistic in vitro assays used later in the assessment. Thyroid effects were not considered in this study because there are a limited number of in silico tools available, mostly for predicting thyroid receptor, which represents only one of several mechanisms involved in thyroid hormone homeostasis (Mullur et al. 2014).

A rich source of data can be found in the EPA's ToxCast database (US EPA). This contains ED data for ~ 1800 chemicals in more than 700 high-throughput assay endpoints that cover a range of high-level cell responses. The US EPA is running an Endocrine Disruption Screening Program (EDSP), with the aim of prioritising chemicals using high-throughput screening methods and computational toxicology approaches to evaluate thousands of chemicals for ED activities. The OECD framework for ED assessment (OECD 2018) requires animal testing at higher levels, where relevant in vitro alternatives are missing e.g. for developmental, reproductive, neurological and immune effects. However, such follow-up testing is not possible for the cosmetics industry especially in the EU due to the complete animal testing ban which came into force in March 2013 (EU 2009). Even for other industries, animal models are not suitable for assessing ED effects due to issues relating to ethics, capacity, speed and relevance (Patisaul et al. 2018). Therefore, chemicals which cannot be evaluated using in vivo method must be assessed using alternative methods e.g. in silico and in vitro methods listed in Levels 1 and 2 of the framework described in the OECD 150 test guideline (OECD 2018).

**Fig. 1:**
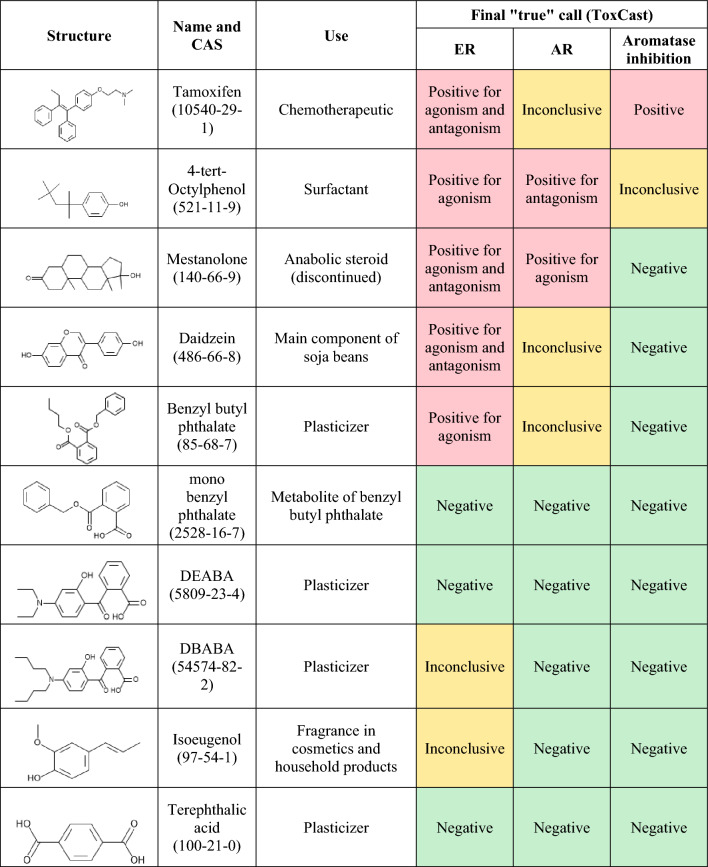
Test chemicals included in the evaluation. The final call with respect to ER and AR effects and aromatase inhibition based on ToxCast data are indicated, along with legacy in vivo data where available. Red denotes positive calls, orange demotes inconclusive calls and green denoted negative calls (color figure online)

We have evaluated a suite of in silico prediction models and in vitro assays reflecting estrogenic, androgenic and steroidogenic effects for their ability to identify the ED properties of ten test chemicals (tamoxifen, 4-tert-octylphenol (4-TO), mestanolone, daidzein, benzyl butyl phthalate (BBP), mono-benzyl phthalate (MBP), 2-(4-(diethylamino)-2-hydroxybenzoyl)benzoic acid (DEABA); 2-[4-(dibutylamino)-2-hydroxybenzoyl]benzoic acid (DBABA), isoeugenol and terephthalic acid, Fig. 1). The chemicals have different uses e.g. drug, plasticizer, fragrance, dietary component (see Fig. 1), but were selected for their differing potentials to cause ED. The selection of the chemicals was first made according to their estrogenic affects, with the aim of including at least 5 known positive chemicals for ER agonism and/or antagonism according to the ToxCast outcome (with in vivo findings, if possible) and which had been tested in OECD Test Guideline assays. Terephthalic acid was included as a true negative control for all endpoints (according to the conclusion on the safety of this chemical by ECHA (ECHA 2017)). There was also one parent–metabolite pair of chemicals (BBP/MBP) selected to evaluate the impact of metabolism.

The in vitro assays evaluated were the YES/YAS assays (Routledge and Sumpter 1996; Sohoni and Sumpter 1998), ER and AR receptor-binding assays (Bowes et al. 2012), (anti)ER- and (anti)AR- “Chemically Activated LUciferase eXpression” (CALUX®) receptor transactivation assays (referred to from here as the “CALUX assay” OECD 2020, 2021)), in vitro H295R steroidogenesis assay (OECD 2022), and a recombinant enzyme aromatase activity inhibition assay (Ji et al. 2014). Five of the chemicals were tested blinded in the CALUX assays (tamoxifen, mestanolone, daidzein, MBP and terephthalic acid). The methods were all conducted in the absence of a metabolic supplement to determine the ED potential of the parent test chemicals. However, metabolism can have a significant impact on the results of assays and can result in the formation of metabolites that may have a lower or greater ED potential than the parent compound. To address this, we also assessed the impact of liver S9 with cofactors for Phase 1 (NADPH) and Phase 2 (uridine 5'-diphosphoglucuronic acid (UDPGA), 3′-phosphoadenosine-5′-phosphosulfate (PAPS) and glutathione) on the outcome of the ER and AR transactivation assays for one chemical, BBP.

In silico prediction models were evaluated for their ability to detect binding, agonism and antagonism of the ER and AR, and aromatase inhibition. These included Derek, Vega, Case Ultra, Danish (Q)SAR, ADMETLab, Opera, ADMET Predictor and ProToxII (see Online Resource 1 Supplementary Table 1 for the list of models and associated references). These in silico models use a range of computational techniques, such as molecular docking (*i.e.* Endocrine Disruptome (Kolšek et al. 2014)), machine learning-based quantitative structure–activity relationships (QSAR), and expert rules-based systems to predict the potential of a chemical to interact with specific endocrine receptors and activate or inhibit their signalling pathways. Vega, ProToxII, Danish (Q)SAR, ADMETLab, ADMET predictor and Case Ultra all use QSARs to predict the potential ED effects of chemicals. Derek is a rules-based system that uses expert knowledge to predict potential toxicity of chemicals based on their structure (Aiba née Kaneko et al. 2015; Verheyen et al. 2017)). ToxCast data have been implemented to build different prediction models e.g. Opera is an open source platform that uses machine learning-based QSARs and integrated in the EPA’s CompTox Chemistry Dashboard (Mansouri et al. 2018). In addition, (Browne et al. 2015 and Kleinstreuer et al. 2017) have a developed ToxCast Pathway Model that integrates ToxCast high-throughput screening assays into a computational model that can discriminate bioactivity from assay-specific interference and cytotoxicity. The ToxCast Pathway Model provides a value (range from 0 to 1) for AR and ER activity (agonism or antagonism). If any of these values exceed 0.1, then there is a significant interaction. The model is based on the concept of the adverse outcome pathway (AOP), which describes the biological sequence of events that lead from chemical exposure to adverse effects. This model has been shown to be effective at predicting the potential toxicity of chemicals across multiple endpoints, including ED. In a recent study, the model was used to predict the estrogenic and androgenic activity of over 1,200 chemicals, and the results were compared to experimental data. The study found that the model was able to accurately predict the estrogenic and androgenic activity of the chemicals with a high degree of accuracy. The outcomes of the ToxCast Pathway Model were considered to derive the ToxCast final call.

The performance of different in silico models for ED effects tends to be evaluated on single model for a single effect, such as ER or AR binding, agonism or antagonism (normally by the developer), or a limited number of models using multiple chemicals (Weyrich et al. 2022). Multi-organisation collaborations have also evaluated multiple models for a specific effect e.g. ER (Mansouri et al. 2016) and AR (Mansouri et al. 2020) effects. Therefore, we have conducted a side-by-side comparison of multiple different in silico models covering three pathways in ED, namely ER, AR and aromatase inhibition and compared these with ToxCast-derived final calls, as well as with results from in vitro assays. The efficiency of the in silico models was evaluated as a metric for the ability of the in silico model to generate concrete predictions. The efficiency (expressed as a % of the chemicals tested) excludes results which are out of the domain (OAD) of the model, as well as inconclusive results, whereby the more chemicals which are OAD or inconclusive, the lower the efficiency. In vivo data were available for some but not all chemicals; therefore, an evaluation of the performance of the methods was made by comparing the derived overall calls from in silico models with those from the ToxCast database. The final call for each chemical evaluated in the ToxCast assays was made by considering the active-flagged assays and the corresponding cytotoxicity levels, the ToxCast Pathway Model (Browne et al. 2015) and the CERAPP Potency Level (Mansouri et al. 2016) for ER effects and the ToxCast Pathway Model (Kleinstreuer et al. 2017) for AR effects (see Online Resource 1 Supplementary Tables 2, 4 and 6 for the outcome of the ER, AR and aromatase ToxCast assays, additional literature and final calls for each chemical and target).

## Methods

### Test chemicals

Tamoxifen, 4-tert-octylphenol (4-TO), benzyl butyl phthalate (BBP), mono-benzyl phthalate (MBP), 2-[4-(dibutylamino)-2-hydroxybenzoyl]benzoic acid (DBABA), isoeugenol and terephthalic acid were from Sigma-Aldrich, Germany. Mestanolone was from TCI Co. Ltd., 2-(4-(diethylamino)-2-hydroxybenzoyl)benzoic acid (DEABA) was from AmBeed Inc., USA, and daidzein was from Cayman Chemicals, Germany.

### In vitro measurements

Full details of the in vitro assay methods are given in Online Resource 3. The YES/YAS XL (Xtra Lyticase) assay kit from Xenometrix AG (Allschwil, Switzerland) was conducted according to the Supplier’s instructions. Binding to ER and AR was conducted using standard methods for radioligand assays (Kurata et al. 2005; Zava et al. 1979). The CALUX Receptor Transactivation Assays were conducted as described previously (Sonneveld et al. 2005; van der Burg et al. 2013). An additional assay was conducted for BBP tested in the ER and AR CALUX assays with metabolic supplements added (according to (van Vugt-Lussenburg et al. 2018)). The inhibition of aromatase activity was measured using human recombinant CYP19A1 as described previously (Ji et al. 2014). The impact of test chemicals on steroidogenesis was measured according to oestrogen and androgen production by H295R cells, according to the OECD test guideline 456 (OECD 2022).

### In silico prediction models

Multiple in silico tools were used to predict estrogenic, androgenic and steroidogenic effects of the test chemicals, summarised in the Online Resource 1 Supplementary Table 1. The table describes the methods implemented, predicted endpoints (binding, activation: agonist or antagonist, or inhibition e.g. for aromatase), type (standalone or web app), available open-access platform, developer and the link together with the model publications. The tools for ER, AR and aromatase effects e.g. Endocrine Disruptome, employ molecular docking to predict the binding affinity of a molecule of interest to the ER and AR, and being agonist or antagonist (Cheng et al. 2012; Yang et al. 2018). The QSAR-based tools e.g. Vega, Opera, Danish (Q)SAR and ProtoxII can predict binding and activation. ADMET Predictor, developed by SimulationsPlus, predicts binding and the relative binding affinity (%RBA) to 17-oestradiol or 7R-methyl-[3H]-methyltrienolone. Two hybrid models with QSAR and expert knowledge components were also included: Derek Nexus and Case Ultra for binding, agonist and antagonist predictions. In addition, admetSAR was used for aromatase inhibition predictions only. Several of the models are standalone e.g. VEGA, Opera and Case Ultra or web applications e.g. ProtoxII and Endocrine Disruptome. In addition, a suite of open-access models is available e.g. VEGA, Opera, ProToxII, Endocrine Disruptome and Danish (Q)SAR. Of note, some of the test chemicals in this study were parts of the training set for several of the in silico models (VEGA, Danish (Q)SAR, Case Ultra and OPERA and these are denoted in the Online Resource 1 Supplementary Table 1). These in silico models enable the user to retrieve experimental data for the compounds of interest if available, as well as generating predictions for the corresponding endpoint. For the in silico protocol used here, we considered the predictions only and ignored the experimental data from the training set. In some cases, there were differences between the prediction and the experimental data in assignment in the training set.

A final call based on all the in silico models considered (a) whether models predicted a chemical to bind to a receptor together with a positive result for activation/antagonism, since these are inherently linked (this provided a higher confidence in a positive result); and (b) concordance between models i.e. if the majority of models indicated a positive (or negative) result, it was given more weight.

### Overall conduct of in silico and in vitro model data generation

The in silico and in vitro model data were generated completely independently (and by different scientists), whereby the results from one model did not impact the interpretation of another. For the in vitro assays, the chemicals were tested blinded.

## Results

### In vitro measurements

#### YES/YAS assays

**Fig. 2:**
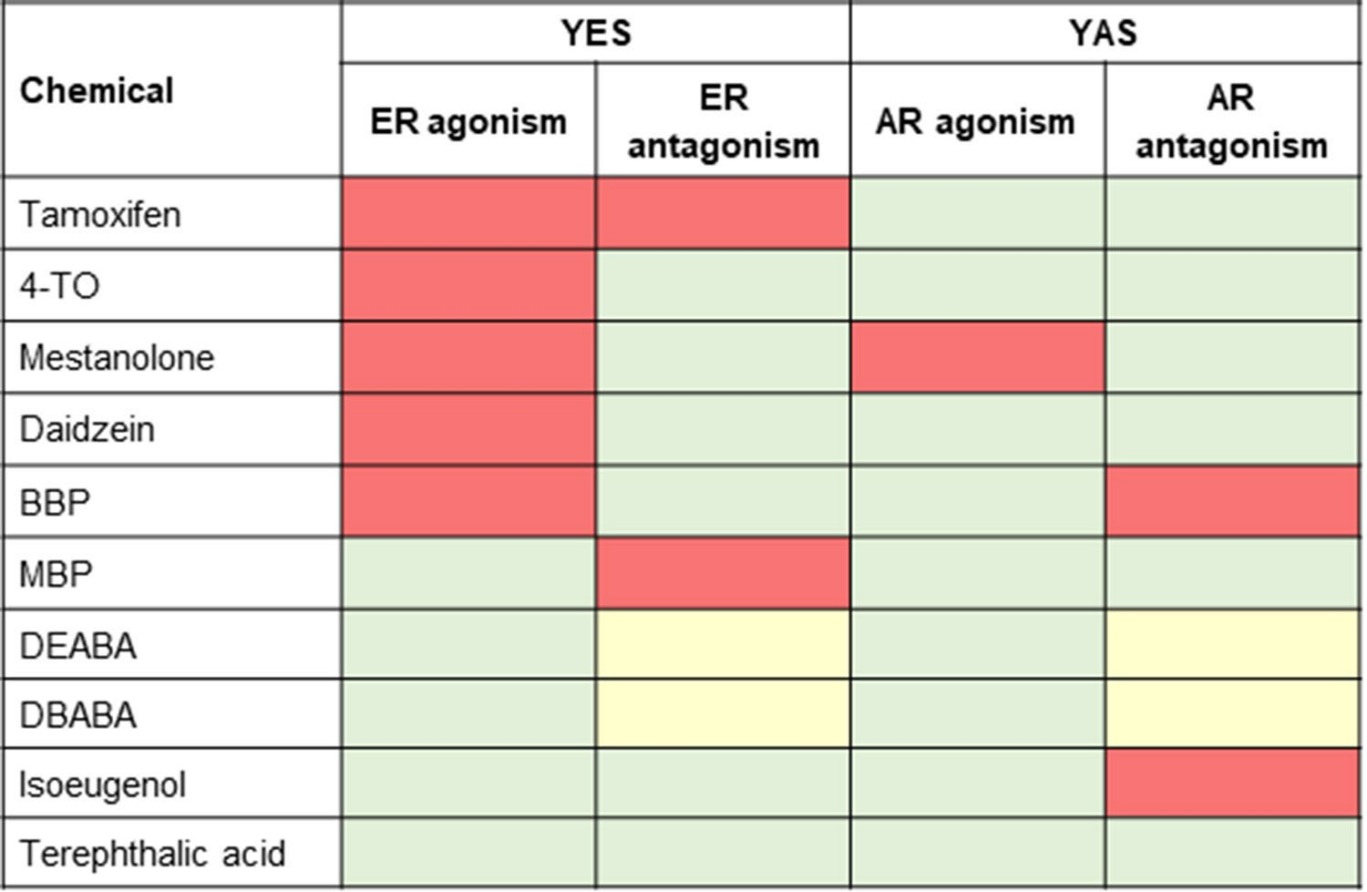
Heatmap of ER and AR agonism and antagonism by the 10 chemicals tested in the YES and YAS assays. Results are from duplicates performed in two independent experiments. Test chemicals which were positive are denoted in light red, chemicals which were negative are denoted in green and chemicals which were inconclusive are in yellow (color figure online)

The concentration curves for the test chemicals with respect to ER agonism/antagonism and AR agonism/antagonism are shown in Online Resource 2 Supplementary Fig. 1 and a summary of the results is shown in Fig. 2. Of the chemicals tested, 5 were positive in the YES assay for ER agonism (tamoxifen, 4-TO mestanolone, daidzein and BBP). One of these, tamoxifen, was also positive in the YES assay for ER antagonism, along with MBP. The decrease in the signals observed in the YES assay for DEABA and DBABA does not show antagonistic effects since both chemicals were positive in both YES and YAS antagonistic assays (which is an indication for non-specific effects stipulated in the supplier’s protocol). For this reason, the results for DEABA and DBABA were inconclusive.

Only one of the chemicals was positive in the YAS assay for AR agonism (mestanolone), whilst 2 were positive in the YAS antagonist assay (BBP and isoeugenol). Tamoxifen and 4-TO were negative in the YAS assay; however, these were toxic at the highest concentrations tested, evident as a decrease in the yeast cell growth.

### ER and AR binding

**Fig. 3:**
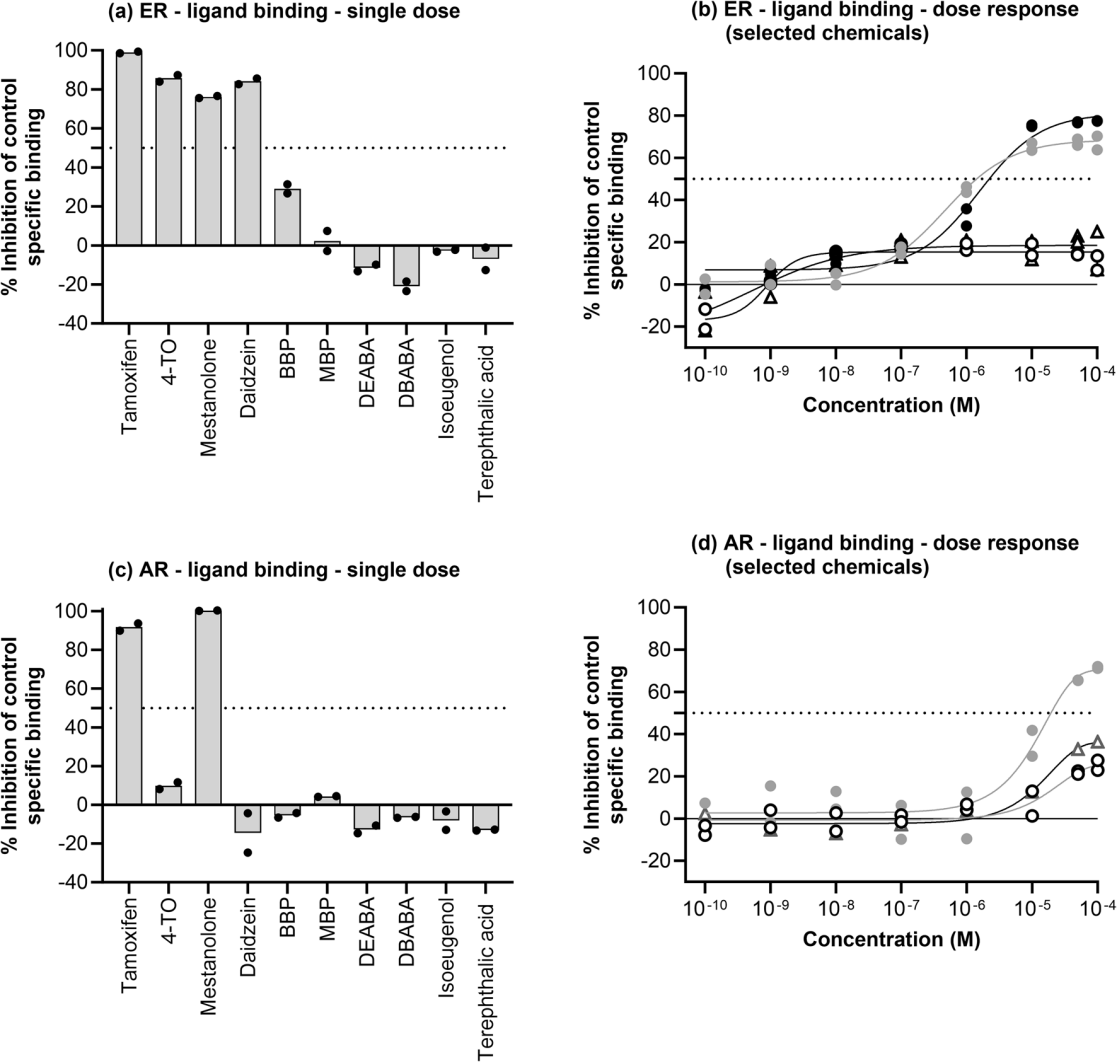
Ligand binding to the ER (**a** and **b**) and AR (**c** and **d**) after incubation with test chemicals using a single (**a** and **c**) and a range of concentrations (**b** and **d**). In (**b**) mestanolone is denoted by black circles, DEABA is denoted by open circles and BBP is denoted by grey circles and MBP is denoted by open triangles. Individual replicate values are shown

In the single-dose screening (10 µM), there were 4 chemicals which resulted in more than 50% inhibition of control specific binding, indicating a significant binding to the ER (tamoxifen, 4-TO, mestanolone and daidzein, Fig. 3a). Low to moderate negative values (less than -25%) for DEABA, DBABA, isoeugenol and terephthalic acid were considered not to be biologically relevant and attributable to variability of the signal around the control level. BBP exhibited weak binding (29% inhibition) and was therefore tested in a follow-up dose–response assay, along with mestanolone (to confirm the positive outcome) and DEABA and MBP (to confirm the negative outcomes) (Fig. 3b). Mestanolone was confirmed to bind to the ER, with IC_50_ and K_i_ values of 1.6 and 0.52 µM, respectively. This assay also indicated that BBP exhibited significant binding to the ER, with IC_50_ and K_i_ values of 0.46 and 0.15 µM, respectively. BBP was therefore classified as positive for ER binding. Of note, the IC_50_ for BBP was lower than was expected from the initial test using 10 µM (Fig. 3a). This was partly attributed to the solubility of this chemical, which is ~ 10 µM and accounts for the plateau reached at this concentration seen in Fig. 3b. Second, the IC_50_ was derived using the concentration at which 50% of the maximum inhibition of BBP was achieved (and not the concentration at which 50% of the maximum inhibition of the positive control was measured, as was measured in the single-dose study). Therefore, fitting the BBP concentration–response curve resulted in a maximum inhibition of 69% (and not 100%) and an IC_50_ of 0.46 µM.

In single-dose screening for binding to the AR, only 2 chemicals (tamoxifen and mestanolone) resulted in more than 50% inhibition of control specific binding (Fig. 3c). All other chemicals did not exhibit significant inhibition of AR binding of the control substance at 10 µM, with the highest inhibition exhibited by 4-TO of 10%. 4-TO, BBP and isoeugenol were tested in a follow-up dose–response assay, which indicated that 4-TO exhibited significant binding, albeit at higher concentrations (IC_50_ and K_i_ values of 27 and 12 µM, respectively) and that BBP and isoeugenol exhibited moderate binding, although the IC_50_ values were > 100 µM) (Fig. 3d).

### Aromatase inhibition

**Fig. 4:**
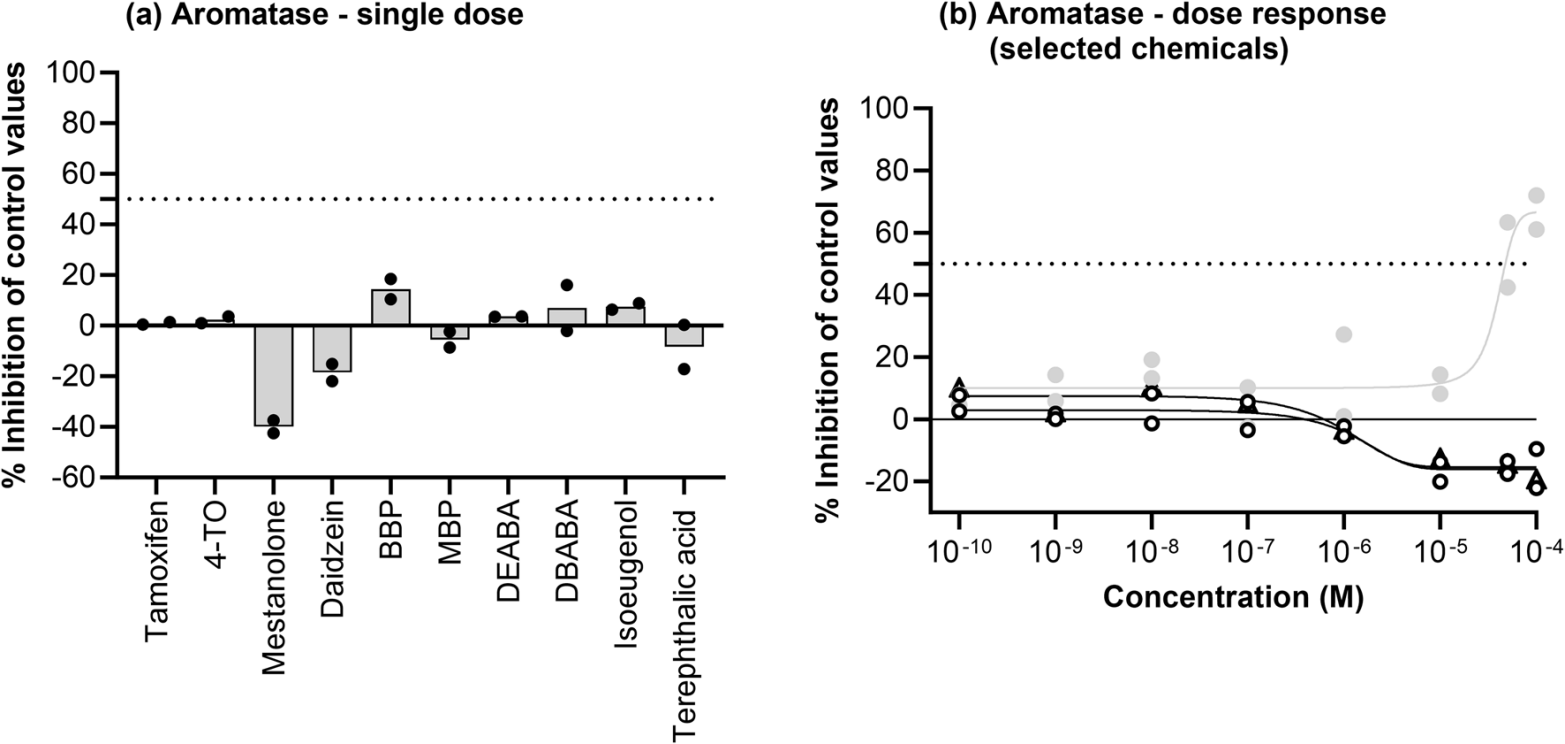
Aromatase inhibition (**a**) by 10 test chemicals incubated at a single concentration of 10 µM and (**b**) by mestanolone (open circles), daidzein (open triangles) and isoeugenol (grey circles) tested in a follow-up dose–response assay. Inhibition was calculated as a % inhibition of control enzyme activity. Individual replicate values are shown

None of the chemicals tested at 10 µM inhibited aromatase activity by more than 50% (Fig. 4a). High negative values (≥ 50%) that are sometimes obtained with high concentrations of test compounds are generally attributable to non-specific effects of the test compounds in the assays; therefore, mestanolone and daidzein, in addition to isoeugenol, were tested in a follow-up dose–response assay (Fig. 4b). This confirmed the lack of binding of daidzein and mestanolone but indicated that isoeugenol inhibited this enzyme at higher concentrations (the IC_50_ was 61 µM). Therefore, isoeugenol was classified as positive for aromatase inhibition.

### CALUX assays

**Fig. 5:**
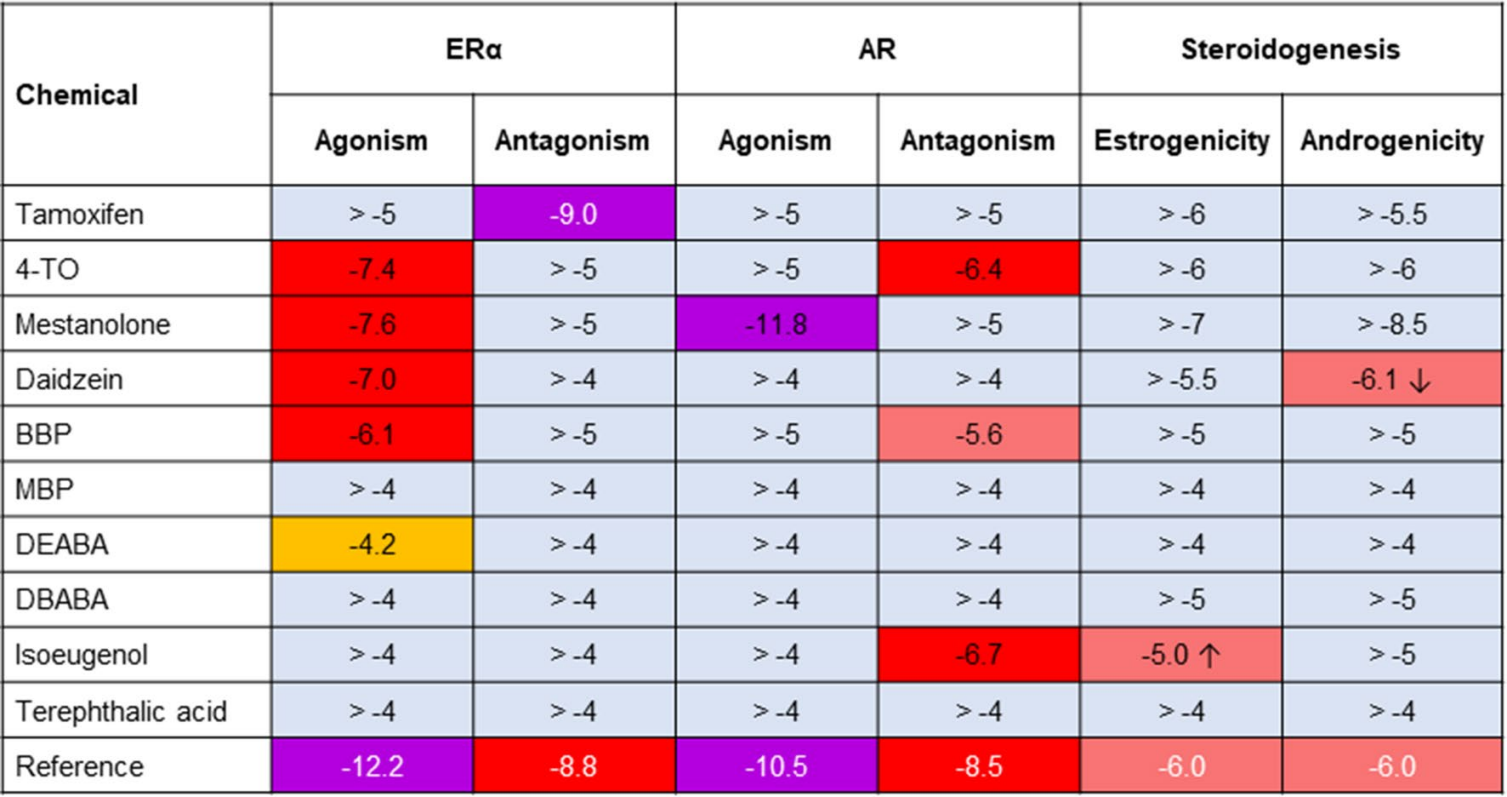
Summary of ER and AR transactivation and steroidogenesis results presented as a heatmap. The LEC values are shown here in Log M; the colour indicates the potency (yellow < orange < red < purple). For comparison, the LEC values of the individual reference compounds of the assays are shown in the bottom row (color figure online)

The concentration curves for the test chemicals with respect to cytotoxicity, ER agonism/antagonism and AR agonism/antagonism are shown in Online Resource 2 Supplementary Fig. 2a, b and c, respectively, and a summary of the results is shown in Fig. 5. There were 4 chemicals that were moderately to highly potent agonists of the ER (4-TO, mestanolone, daidzein and BBP, with Lowest Effective Concentration (LEC) values of 0.04, 0.028, 0.1 and 0.69 µM, respectively) and one chemical which was a weak agonist of the ER (DEABA, LEC was 56 µM). Tamoxifen, which was tested blinded, was identified as a potent antagonist of the ER (LEC was 1.1 nM, as expected since it is the positive control for the assay).

There was only one chemical that was an AR agonist, namely mestanolone, which exhibited potent activation of the receptor (LEC was 1.6 pM). This was expected since it is the positive control for the assay (although it was tested blinded in this study). There were 3 AR antagonists with weak potency (BBP, LEC was 2.4 µM) to moderate potency (4-TO and isoeugenol with LEC values of 0.44 and 0.29 µM, respectively).

### H295R Steroidogenesis assay

The concentration curves for the test chemicals with respect to oestrogen and androgen production are shown in Online Resource 2 Supplementary Fig. 2d and a summary of the results is shown in Fig. 5 (right-hand columns). There were only two chemicals that affected hormone production in H295R cells. Isoeugenol increased oestrogen production by the cells by 1.5-fold of control at the highest concentration tested (100 µM, the LEC was 10 µM) and daidzein decreased androgen production to 0.06-fold of control at the highest concentration tested (100 µM, LEC was 0.77 µM).

### Impact of metabolism on the CALUX assay

**Fig. 6:**
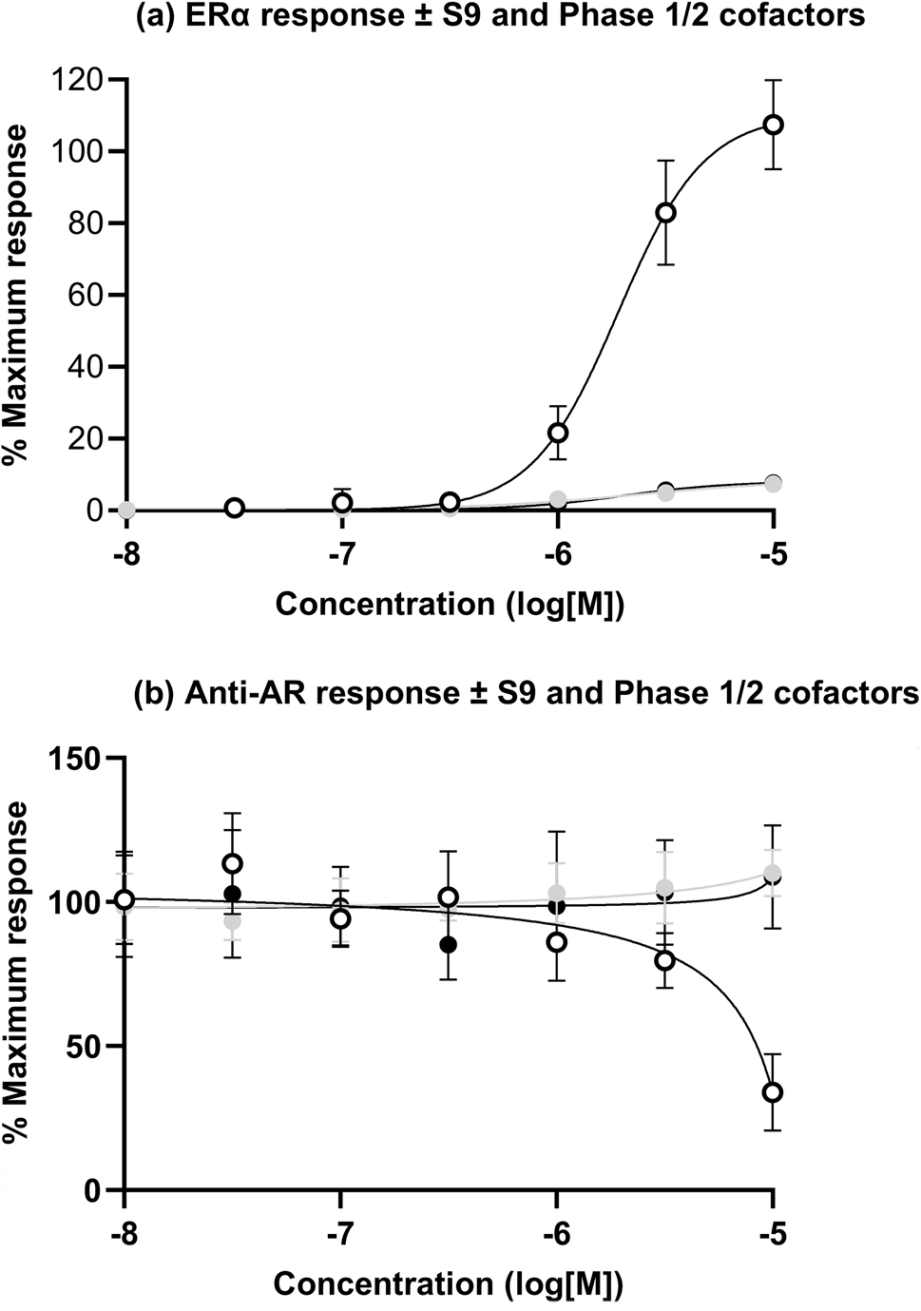
Impact of including liver S9 and Phase 1 and 2 cofactors on the outcome of the ER (**a**) and anti-AR (**b**) CALUX assays for BBP. Incubations were conducted in the absence of liver S9 (open circles), S9 + Phase 1 cofactors (grey circles) and S9 + Phase 1 + 2 cofactors (black circles), values are a mean ± SD, *n* = 6)

The impact of including liver S9 and Phase 1 and 2 cofactors on the outcome of the ER and AR CALUX assays was evaluated using BBP (Fig. 6), as MBP was the corresponding metabolite tested in all assays. In the absence of S9, there was a concentration-dependent activation of ERα by BBP (Fig. 6a); however, activation was abolished in the presence of S9 with the phase 1 cofactor, NADPH, and in the presence of NADPH and phase 2 cofactors (reduced glutathione, PAPS and UDPGA). As for ERα activation, the AR antagonism exhibited by BBP in the absence of S9 and cofactors was abolished by co-incubating with S9 and both cofactor supplements (NADPH only or NADPH and Phase 2 cofactors) (Fig. 6b).

### In silico predictions

The outcomes of the individual in silico predictions of the effects of the test chemicals on the ER, AR and aromatase activities are shown in Online Resource 1 Supplementary Tables 3, 5 and 7, respectively. There were multiple in silico methods used to predict estrogenic effects (18 models) and androgenic (17 models) effects, whilst only 4 were used to predict effects on aromatase activities (as indication of steroidogenic effects). The overall calls based on all in silico methods are listed in the tables, along with the final call from the ToxCast assays.

#### Estrogenic effects

**Fig. 7:**
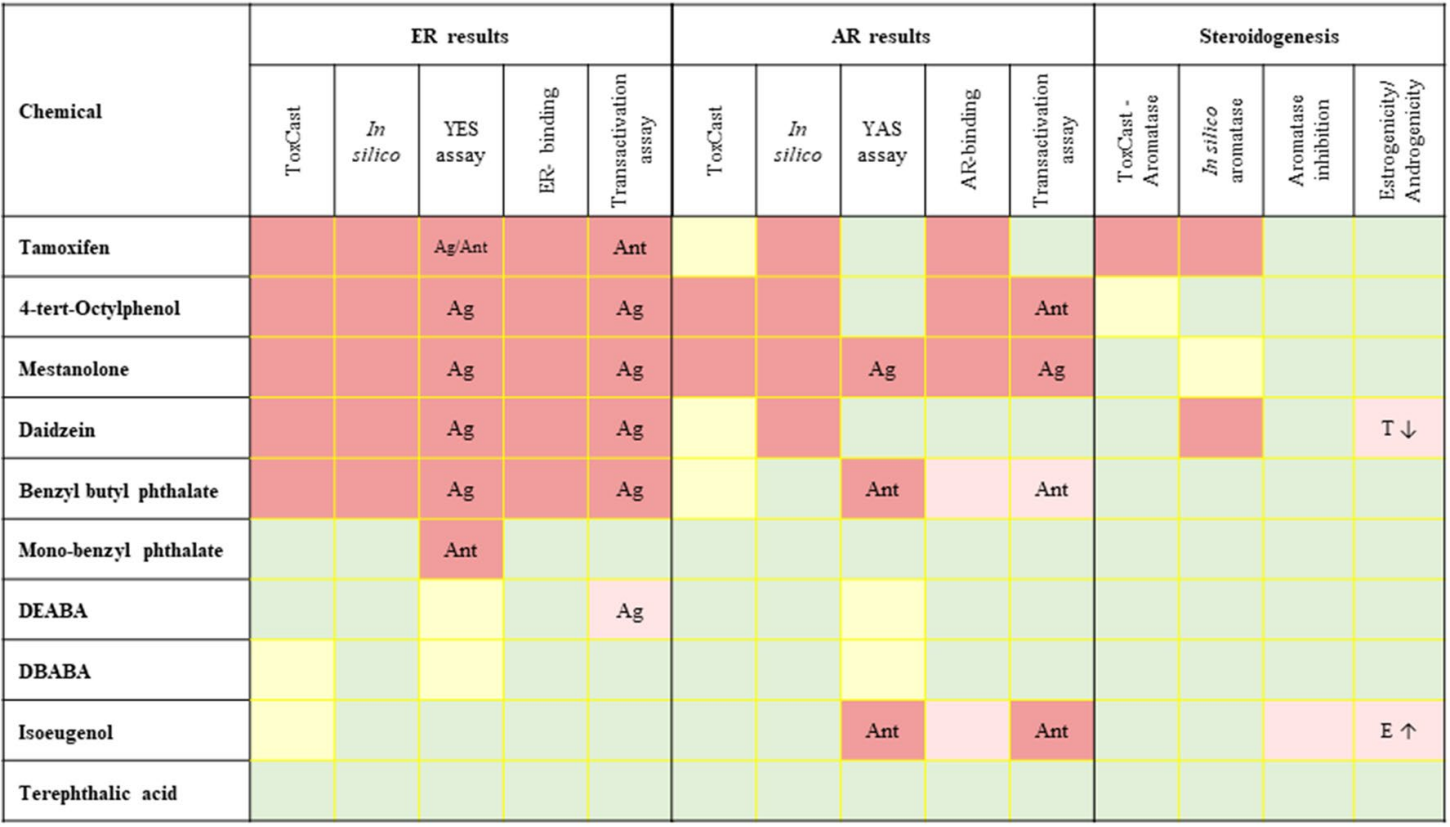
A summary of the results from in silico and in vitro methods to detect potential estrogenic, androgenic and steroidogenesis effects of 10 model chemicals. Results are colour-coded: light red indicates a positive result, pink indicates a weak positive result, yellow indicates an inconclusive result and green indicates a negative result. Ag = agonist, Ant = antagonist, E ↑ = estrogenicity (increase in oestrogen production) and T ↓ = androgenicity (decrease in testosterone production) (color figure online)

The final call from the in silico predictions indicated that 5 chemicals (tamoxifen, BBP, daidzein, 4-TO and mestanolone) were classified as having estrogenic effects (Fig. 7). These chemicals were predicted to be positive by the majority of the 18 in silico models evaluated, regardless of whether the model predicted agonistic or antagonistic effects (Online Resource 1 Supplementary Tables 3). This provided high confidence in the in silico final call. A notable exception was with respect to Endocrine Disruptome models, which predicted 9 out of 10 chemicals to be negative (thus incorrectly predicting 4 out of 5 of the chemicals classified as positive). Of note, whilst daidzein is classified according to the ToxCast outcome as being an ER agonist and antagonist, 4-TO is classified as being an ER agonist only, indicating that the predictions by two models for ER antagonism, Case Ultra and Opera, were less precise with respect to the type of action on the ER. The Opera and Danish (Q)SAR models indicated that tamoxifen binds to the ER but did not activate and/or inhibit it. The same was true for the Vega model for BBP. The remaining 5 chemicals were classified as negative based on all in silico model predictions, with the majority of the models (10–15 out of 18 models) indicating a lack of ER effects. Several of the test chemicals were out of the domain of the models for ER effects (mestanolone for the Vega ER activation model, MBP for the Vega ER-binding model, DBABA for the ADMET prediction model and isoeugenol for the Vega activation, Danish (Q)SAR ER activation and Opera ER-binding models).

The efficiency and the predictivity of ER effects by the individual models compared to the final ToxCast classification are shown in Table 1. Of note, the number of chemicals included in the calculations of the % of correct calls was lower when the ToxCast result was inconclusive (for ER effects by DBABA and isoeugenol the final calls were inconclusive) and when the outcome of the in silico model was inconclusive. Most of the models (13 out of 18) were 100% efficient in giving a distinct prediction on the ER effects of the 10 test chemicals studied. The remaining models also showed a high efficiency (75–88%) or a moderate efficiency (63% for ADMETLab ER). There were three models, Danish (Q)SAR ER binding, Opera ER binding and ProToxII LBD, which correctly predicted the outcome of 8 chemicals included in the analysis. ADMET predictor exhibited the lowest percentage of correct calls (50%) and the remaining models predicted between 63 and 88% of the calls.

**Table 1:** Evaluation of in silico models

In silico model		ER		AR		Aromatase	
		% Correct calls	% Efficiency	% Correct calls	% Efficiency	% Correct calls	% Efficiency
Derek		63	100	71	100	–	–
Vega	Binding	75	100	–	–	–	–
	Activation	86	88	86		–	–
	Aromatase	–	–	–	–	78	100
Case Ultra	Binding	83	75	–	–	–	–
	Agonist	88	100	67	43	–	–
	Antagonist	75	100	57	100	–	–
Danish	Binding	100	100	100	57	–	–
	Activation/Agonist	88	100	100	86	–	–
	Antagonist	–	–	100	57	–	–
ADMET Lab	ER/AR	60	63	67	86	–	–
	LBD	88	100	83	86	–	–
	Aromatase	–	–	–	–	100	89
Opera	Binding	100	100	80	71	–	–
	Agonist	75	100	100	86	–	–
	Antagonist	63	100	67	86	–	–
ADMET predictor	Binding	50	100	86	100	–	–
Endocrine Disruptome	Agonist	57	88	100	71	–	–
	Antagonist	71	88	0	14		–
ProToxII	ER/AR	88	100	86	100	–	–
	LBD	100	100	86	100	–	
	Aromatase	–	–	–		89	100
admetSAR	Aromatase	–	–	–		33	100

Values are the percentages of correct calls for ER and AR agonism and/or antagonism and aromatase inhibition by different in silico models when compared to the final ToxCast call. There were 8, 7, and 9 chemicals included in the comparison of ER, AR, and aromatase effects, respectively (chemicals with inconclusive calls from ToxCast were excluded). Models for which the chemicals were out of the applicability domain were given a value of zero in the calculation

#### Androgenic effects

The final call from the in silico predictions indicated that 4 of the 5 chemicals causing ER effects were also classified as having androgenic agonistic and/or antagonistic effects (tamoxifen, 4-TO, mestanolone and daidzein) (Fig. 7). BBP, MBP, DEABA, DBABA, isoeugenol and terephthalic acid were negative for androgenic effects. In comparison with the ER predictions, there were more inconclusive outcomes from the in silico prediction of AR effects, with each chemical being inconclusive in 1–3 models.

The predictivity of AR effects by the individual models is shown in Table 1. There were 6 out of 17 models that were 100% efficient in giving a prediction on AR effects of all ten test chemicals (Derek, Vega activation, Case Ultra antagonist, ADMET predictor, ProToxII AR and ProToxII LBD). Several other models also showed a high efficiency between 71 and 86%, whilst three only returned distinct predictions for fewer than 60% of the test chemicals (Case Ultra agonist, Danish (Q)SAR binding, Danish (Q)SAR antagonist and Endocrine Disruptome. Predictions from several models (the three Danish (Q)SAR models, Opera agonist and Endocrine Disruptome) were correct in 100% of the cases for which they returned a distinct prediction. ADMET predictor, Opera and Vega correctly identified the outcome of a higher number of chemicals for AR effects (86%) compared to ER effects for which they returned a distinct prediction. The Endocrine Disruptome model exhibited the highest inconclusive results (i.e. the % efficiency was only 14%) and the lowest % of correct calls (none of the calls were correct).

#### Aromatase inhibition

The final call on the in silico predictions indicated that 2 chemicals (tamoxifen and daidzein) were classified as inhibiting aromatase activity. There were conflicting results for mestanolone, with 2 models predicting a positive result and 2 predicting a negative result. The Vega model, which resulted in a positive prediction for mestanolone, used this chemical in its training set, whilst the admetSAR model predicted this chemical to be positive, with a good confidence of 87% but not using this chemical in its training set. Whilst the AdmetLab model predicted mestanolone to be negative with low confidence, the ProToxII model predicted a negative result with high confidence. As a result, the overall call for this chemical was inconclusive.

The predictivity of effects on aromatase by the individual models is shown in Table 1. Whilst the efficiency of all 4 aromatase models was very good (89–100%), three of the four models exhibited a higher percentage of correct calls for the 9 chemicals, indicating that there is a high confidence in the results from these models. The admetSAR model showed oversensitivity that resulted in 6 incorrect calls which were false positives.

## Discussion

EU regulations and others require the investigation of chemicals for potential ED properties to ensure the safety of human health and the environment. The conduct of a NGRA is an exposure-led, hypothesis-driven approach which is being implemented to enable the registration of cosmetic ingredients without the generation of new data in animals (Alexander-White et al. 2022; Dent et al. 2021). Whilst much progress has been made in recent years with respect to the development of in vitro and in silico approaches used in an NGRA, more effort is needed to demonstrate their use in a regulatory context (Dent et al. 2021). Therefore, this study evaluated a suite of in silico and in vitro methods for their potential to detect potential estrogenic, androgenic and steroidogenesis effects of 10 model chemicals, as part of the hypothesis forming and MoA investigation steps of an NGRA. Exposure was not considered since these models are intended for in initial screening; therefore, the concentrations and the points of departure were not compared with predicted/measured internal exposures to obtain margins of safety (this would be conducted in the later tiers of the safety assessments. A summary of the outcome of all the results is shown in Fig. 7. It should be noted that, with one exception, all in vitro assays were conducted without a metabolic supplement; therefore, the ED potential of the test chemical themselves (and not their metabolites) was measured.

## Comparison of results from in vitro assays

The YES/YAS assays are time- and cost-effective assays using a simple readout, which is used to detect both agonistic and antagonist effects of test chemicals on the ER and AR. The YES assay correctly identified tamoxifen as both an agonist and antagonist of the ER (Dhingra 1999), whilst the CALUX ER assay only detected antagonism of this chemical. Four additional chemicals which were indicated to be ER agonists in the CALUX assay were also positive for ER agonism in the YES assay. Thus, there was a good concordance of this assay with other in vitro (and in silico and in vivo for chemicals for which these data were available) models for these 5 chemicals, indicating that this can be used for screening early in an NGRA before moving to OECD test guideline assays, depending on the biological plausibility. There was only one false positive outcome in the YES assay (in which MBP was positive for ER antagonism) and indicating that this assay can be over-sensitive. For isoeugenol, there was difference in the YAS assay outcome compared to the ToxCast classification for AR antagonism; however, isoeugenol exhibited weak binding to the AR and was positive in the CALUX transactivation assay for AR antagonism, indicating the parent chemical exhibits AR antagonism (but potentially, the metabolites do not). This attribute can be considered beneficial in having confidence in a negative outcome, as well as representing a worst-case scenario during the screening of many chemicals. Two chemicals were inconclusive in the antagonist YES/YAS assays, DEABA and DBABA due to non-specific effects; however, this result is easy to identify and can be followed up using an alternative assay. The negative results for tamoxifen and 4-TO YAS assay were due to cytotoxicity occurring at the highest concentrations tested (masking any ER or AR effects); therefore, we recommend a follow-up assay using a lower concentration range with closer spacing between the concentrations to determine whether a positive concentration-dependent response can be observed at non-cytotoxic levels.

The outcomes from the ER and AR binding assays correlated well with those from the ER and AR CALUX assays using this selection of chemicals. This was more apparent for the AR outcomes, which showed that AR binding was linked to AR agonism by mestanolone and AR antagonism by 4-TO, BBP and isoeugenol, whereas chemicals that did not bind to the AR (daidzein, MBP, DEABA, DBABA and terephthalic acid) were negative in the AR transactivation assay. Our study indicates that the inclusion of follow-up dose–response assays is recommended to confirm the outcome of the single concentration screening of ER and AR binding using radio-ligands. This was demonstrated for the weak binding of BBP in the ER-binding assay and the significant binding 4-TO and moderate binding of BBP and isoeugenol in the AR binding assays.

Aromatase represents only one enzyme involved in steroidogenesis (the conversion of androgens to oestrogens); therefore, the aromatase assay detects effects on 17β-oestradiol production only. There were only two chemicals classified based on ToxCast data as positive (tamoxifen) or inconclusive (4-TO) for aromatase inhibition, neither of which were positive in the aromatase inhibition or steroidogenesis assays performed in this study. The inconclusive result for 4-TO from the ToxCast panel indicated that the inhibition of aromatase was only borderline (the efficacy was 31.35%); therefore, the negative result from the aromatase inhibition and H295R steroidogenesis assays provides an additional WoE that this is negative for steroidogenesis. There are two possible reasons for the discrepancy between the ToxCast classification and the negative outcome of the aromatase inhibition assays for tamoxifen. First, investigations into the aromatase inhibitory action of tamoxifen using recombinant and placental microsomal preparations show that its metabolites, endoxifen and N-desmethyl-tamoxifen, are potent inhibitors, whilst tamoxifen itself exhibited no appreciable inhibition (Lu et al. 2012). Second, the “TOX21_Aromatase_Inhibition” assay used to classify tamoxifen detects not only aromatase inhibition but also ER agonism or antagonism. It is a cell-based assay in which MCF-7 cells are incubated with the test chemical for 24 h before measuring aromatase activities indirectly via a luciferase-based reporter endpoint which is dependent on the binding of the oestradiol–ER complex to the oestrogen response element. A decrease in luciferase activity can occur as a result of direct inhibition of aromatase (preventing the production of oestradiol) or by preventing oestradiol from binding to the ER and thus the binding to the oestrogen RE which initiates the production of luciferase (Lui et al. 2008). This means that a positive result from this cell-based assay for anti-oestrogens like tamoxifen is likely to be due to inhibition of oestradiol binding and not to direct aromatase inhibition. Therefore, the result from our study using recombinant enzymes is in accordance with the reported lack of direct aromatase inhibition by the parent chemical, tamoxifen, and that additional incubations for this assay using recombinant enzyme should be conducted with a metabolic supplement to detect or rule out inhibitory potential of the metabolites of a test chemical.

Isoeugenol was the only chemical which was positive for estrogenicity and aromatase inhibition in the current study but classified as negative for these effects according to the ToxCast data. Isoeugenol is an isomer of eugenol, which is reported to cause cytotoxicity in MCF-7 cells via an ERα-dependent mechanism leading to apoptosis (Nafie et al. 2022); therefore, the two chemicals may share a common mechanism of action which is detected by the estrogenicity assay. Whilst isoeugenol inhibited aromatase, the potency was low, with an IC50 of 61 µM, and it was negative in the single-dose screening assay. These findings suggest that the results for this chemical are perhaps specific to MCF-7 cells (not to healthy non-immortalised cells) and only occur at high concentrations.

## Metabolism

There was a pair of test chemicals which represented a parent and its corresponding metabolite, namely, BBP and MBP, respectively. ER and AR results from binding and transactivation assays showed that when BBP was positive in an assay, MBP was negative, indicating that metabolic conversion from BBP to MBP resulted in detoxification. This was also supported by additional incubations in which the ER agonism and AR antagonism of BBP were abolished when rat liver S9 was added to the CALUX assays. In this example, the presence of Phase 1 xenobiotic-metabolising enzymes was sufficient to decrease the potency of BBP, but there may be cases when phase 1 pathways lead to activation and subsequent phase 2 pathways lead to detoxification. Therefore, phase 2 cofactors could be added to the incubation so that the assay can reflect both phase 1 and 2 metabolism. The example shown here employed reduced glutathione, PAPS and UDPGA as Phase 2 cofactors; however, further studies are needed to optimise the cofactor mix to include other pathways e.g. N-acetyl transferases, and the best concentration of each to best reflect the balance of metabolism observed in intact hepatocytes (as used by others for metabolism studies Eilstein et al. 2020; Lester et al. 2021)).

## Assessment of individual in silico models

One of the advantages of in silico prediction models is their speed, efficiency and cost-effectiveness, compared to traditional in vivo and in vitro methods (Valerio 2009). They can also be used to screen large numbers of chemicals quickly and identify potential ED chemicals before further testing. Therefore, this study also included a comparison of the performance of individual models to determine whether one or two could be prioritised for use in a safety assessment. It is acknowledged that the number of chemicals used to reflect the predictive capacity is small; however, this enabled their evaluation in multiple in silico models and a side-by-side comparison (also with in vitro data generated in this study). To evaluate the predictive capacity of individual in silico models, the results from these were compared with an overall call from ToxCast in vitro assays. This was because in vivo data are not available for all chemicals tested here; however, the ToxCast-based classifications were confirmed with legacy in vivo data where possible (denoted in Online Resource 1 Supplementary Table 2 for ER effects and Online Resource 1 Supplementary Table 4 for AR effects).

Based on these results, most of the in silico models evaluated predicted ER and AR effects well, especially ER binding and agonism (compared to antagonistic effects). These findings are in accordance with those of the multi-model comparisons conducted by others, in which the average predictive accuracy of models for ER and AR effects (not used here) were 90% and 80%, respectively (Mansouri et al. 2016, 2020). There were three models that correctly predicted ER effects of all 8 chemicals with distinct final call from ToxCast (Danish (Q)SAR Binding, Opera Binding and ProToxII LBD), whereas efficiency of the 15 models used to predict AR effects was below 100% due to several inconclusive calls. The minimum number of correct calls by a single model for ER and AR effects was 50% (by ADMET predictor) and 57% (Case Ultra antagonist), respectively, and the mean number of correct calls for both ER and AR effects by all models was 78% and 79%, respectively. In general, models which predicted ER effects well also predicted AR effects well. When the number of correct calls for both ER and AR effects were combined (Online Resource 1 Supplementary Table 8), the Danish (Q)SAR (all 3 models), Opera (binding and agonist models), ADMET Lab LBD and ProToxII (all 3 models) models demonstrated the best overall performance (with a combined correct call of 86–100%), whilst Endocrine Disruptome demonstrated the lowest performance (with a combined correct call of 36%). With respect to aromatase inhibition, the admetSAR model resulted in 6 incorrect calls which were false positives, indicating that this model is relatively oversensitive in detecting this endpoint in this chemical scenario.

In silico prediction models rely on accurate and reliable information on the chemical structures, biological endpoints and toxicological mechanisms. These models also reflect the quality and relevance of the training data sets used to develop them, which define their applicability and accuracy of the predictions. The underlying data sets should be large enough to expand the chemical space of interest and improve the performance of the model. In the current study, several of the chemicals of interest were also in the training set of a few of the models (denoted in Online Resource 1 Supplementary Tables 3, 5 and 7). Whilst this could be perceived to add bias to the evaluation of a model, it didn’t necessarily lead to a better predictivity for that chemical. For example, BBP is an ER agonist but not an antagonist and was used in the training sets for the Case Ultra Agonist and Antagonist models. Whilst Case Ultra correctly identified it as an agonist, it incorrectly also identified BBP as an antagonist. Another notable observation was that all 16 models predicted 4-TO to be positive for ER effects, even though this is classified as being an ER agonist only. Whilst this suggests the two models for ER antagonism, Case Ultra and Opera, were less precise, the results using them would still indicate that this chemical has the potential to cause ER effects.

The efficiency was used as a metric to reflect the ability of the in silico model to generate concrete predictions. This is of importance since models that have a limited applicability domain or result in multiple inconclusive results are limited in their practical applicability. The efficiency of ER and aromatase models was very good, with most models being able to generate concrete calls for 88–100% of chemicals. The efficiency of the AR models was generally slightly lower than for ER models, but most were still between 71 and 100% efficient. Notable outliers were the Case Ultra AR agonist model and the Endocrine Disruptome model, for which the efficiencies were only 43% and 14%, respectively. Inconclusive results were obtained for several chemicals for ER and AR effects and as such, would trigger follow-up investigations in a safety assessment. The results from the in silico models can then be used to guide follow-up in vitro experiments to complete the risk assessment (in silico-based hypothesis-driven testing) for ER, AR and steroidogenesis. The in vitro assays can be used to confirm the predicted activities and, if active, derive a potency value (IC50, IC10 etc.) to be compared with predicted internal exposure (using PBPK modelling) to perform an exposure-driven risk assessment, similar to those conducted by others (Alexander-White et al. 2022; Bury et al. 2021; Hewitt et al. 2022; Ouedraogo et al. 2022).

## Overall call and comparison of in silico final calls and results from in vitro models with ToxCast classifications

The outcomes for the 10 chemicals from the panel of in silico and in vitro ER models in our study exhibited a good concordance (with the exception of MBP in the YES assay) and generally correlated with their classifications of ER effects based on ToxCast assays. For example, tamoxifen, 4-TO, mestanolone, daidzein and BBP were all positive for ER effects based on the overall call from in silico models and the outcome of individual in vitro assays, moreover, all were in accordance with positive ER classification based on ToxCast assays. All outcomes based on the overall call from in silico models and most of the calls from the in vitro assays indicated MBP, DEABA, DBABA, isoeugenol and terephthalic acid to be negative. DEABA was a weak agonist in the ER CALUX assay and only reached the 10% threshold at the highest concentration tested, 100 µM, which would be unlikely to be observed in vivo. The ToxCast classification for DBABA and isoeugenol were inconclusive but mainly due to borderline efficacy and weak agonist/antagonist effects in some but not all assays in the ToxCast assay panel. Based on all data, including ToxCast, these 5 chemicals could be concluded to be negative for ER effects, especially if plasma concentrations were incorporated into the interpretation.

There was only one chemical that was classified as positive for AR effects by all models (specifically, an AR agonist), namely, mestanolone. This could be expected since it is a known AR agonist (van der Ven et al. 2003; Zakár et al. 1986)) and is used as reference chemical for the AR CALUX assay (OECD 2020). Inconclusive classifications of AR effects from ToxCast assays were reflected as both positive and negative outcomes from the in silico and in vitro methods. For example, tamoxifen was positive based on the overall call from in silico predictions and AR binding assays but negative in the YAS and CALUX transactivation assays. Likewise, daidzein and BBP were classified as inconclusive from the ToxCast assays and exhibited mixed results from the panel of in silico and in vitro AR models.

An interesting finding was observed for isoeugenol, which was indicated to be negative for AR effects based on the overall call from the ToxCast data and in silico predictions but was positive for antagonism in all three in vitro AR assays. This chemical was also positive in one ToxCast assay but the effect was weak and only occurred at the highest concentration tested (which was also observed in the YAS assay). According to the ToxCast Pathway model, which considers the MoA in the interpretation of the assay that integrates 18 ToxCast high-throughput screening assays results were into a computational model that can discriminate bioactivity and cytotoxicity (Kleinstreuer et al. 2017), the interaction of isoeugenol with the AR is predicted to be insignificant, which is why the overall conclusion from the ToxCast assays for our evaluation was given as negative. This finding is supported by in vivo data from developmental toxicity studies in rats given isoeugenol at 250–1000 mg/kg/day, which showed some maternal toxicity at all does but no treatment-related effects on foetuses up to 500 mg/kg/day (George et al. 2001). The difference between the results from ToxCast and in silico with those from the in vitro assays is likely to be due to the impact of metabolism, since OECD test guideline compliant in vitro assays indicated that isoeugenol exhibits AR antagonism which is decreased by metabolism (Park et al. 2021). This underlines the importance of considering metabolism in the ED screening assays.

There were 5 chemicals for which the final calls for steroidogenesis from ToxCast and all the in silico models, together with the in vitro assays were all negative (BBP, MBP, DEABA, DBABA and terephthalic acid). Therefore, there is a high confidence in the final negative call for these chemicals. The final overall call for steroidogenesis effects of 4-TO and mestanolone could also be concluded to be negative, since both in vitro assays were negative and either the ToxCast or in silico results were also negative. There were three chemicals with opposing outcomes using in silico and in vitro assays (tamoxifen, daidzein and isoeugenol); therefore, these would be considered to be inconclusive outcomes and would warrant follow-up investigations to understand the differences in the call and whether the effect would occur at human exposure levels.

## Conclusions

This study highlights the importance of using a combination of in silico prediction models and in vitro assays to evaluate the ED potential of chemicals. In silico prediction models for ED have the potential to be valuable tools for risk assessment and chemical screening. This study demonstrates that based on the 10 chemicals, the in silico models used predicted ER and AR effects well, especially ER binding and agonism, indicating that their use is a good start to an assessment of a new chemical in an NGRA. Danish (Q)SAR (all 3 models), Opera (binding and agonist models), ADMET Lab LBD and ProToxII (all 3 models) models demonstrated the best overall performance for ER and AR effects. Inhibition of aromatase was best predicted by the Vega, AdmetLab and ProToxII models.

In silico prediction models and YES/YAS assays can be used for initial screening and guidance for further investigation in the early tier of an NGRA, and depending on the level of biological plausibility that is needed, further testing can be conducted e.g. using receptor-binding or CALUX and H295R steroidogenesis assays. The results can then be complemented by other testing approaches, such as additional in vitro mechanistic assays and legacy data from in vivo studies, to ensure comprehensive and accurate assessments of ED potential. Factors, such as metabolism, pharmacokinetics and tissue distribution, may influence the potency and efficacy of chemicals and their interactions with receptors in vivo. Therefore, in addition to characterising ED effects, exposure-driven NGRAs according to the consumer use scenarios will help to refine the assessment further.

In conclusion, results using in vitro assays and in silico models for effects on ER and AR were comparable. The results from in vitro assays and in silico assessments should be used in combination and interpreted with other information sources to assess the potential ED effects of chemicals. As the ED system is more complex than is possible to cover using the available NAMs evaluated in this study, further studies with more chemicals and different assays (e.g. reflecting different pathways and mechanisms, such as the thyroid pathway) covering broader chemical and biological spaces may be necessary to detect and characterise potential ED effects of chemicals.

### Supplementary Information

Below is the link to the electronic supplementary material.

Supplementary file1 (XLSX 89 KB)

Supplementary file2 (PDF 614 KB)

Supplementary file3 (DOCX 41 KB)

## Data Availability

All data underlying the results are available as part of the article and no additional source data are required.

## References

[CR1] Aiba née Kaneko M, Hirota M, Kouzuki H, Mori M (2015) Prediction of genotoxic potential of cosmetic ingredients by an in silico battery system consisting of a combination of an expert rule-based system and a statistics-based system. J Toxicol Sci 40(1):77-98. 10.2131/jts.40.77 10.2131/jts.40.77 25743748

[CR2] Alexander-White C, Bury D, Cronin M (2022). A 10-step framework for use of read-across (RAX) in next generation risk assessment (NGRA) for cosmetics safety assessment. Regul Toxicol Pharmacol.

[CR3] Amir S, Shah STA, Mamoulakis C, et al. (2021) Endocrine disruptors acting on estrogen and androgen pathways cause reproductive disorders through multiple mechanisms: a review. Int J Environ Res Public Health 18(4) 10.3390/ijerph18041464 10.3390/ijerph18041464 PMC7913912 33557243

[CR4] Bowes J, Brown AJ, Hamon J (2012). Reducing safety-related drug attrition: the use of in vitro pharmacological profiling. Nat Rev Drug Discovery.

[CR5] Browne P, Judson RS, Casey WM, Kleinstreuer NC, Thomas RS (2015). Screening chemicals for estrogen receptor bioactivity using a computational model. Environ Sci Technol.

[CR7] Bury D, Alexander-White C, Clewell HJ (2021). New framework for a non-animal approach adequately assures the safety of cosmetic ingredients—a case study on caffeine. Regul Toxicol Pharmacol.

[CR8] Cheng F, Li W, Zhou Y (2012). admetSAR: a comprehensive source and free tool for assessment of chemical ADMET properties. J Chem Inf Model.

[CR9] Dent MP, Vaillancourt E, Thomas RS (2021). Paving the way for application of next generation risk assessment to safety decision-making for cosmetic ingredients. Regul Toxicol Pharmacol.

[CR10] Dhingra K (1999). Antiestrogens—Tamoxifen, SERMs and Beyond. Invest New Drugs.

[CR11] ECHA et al. (2018) European Chemicals Agency (ECHA) and European Food Safety Authority (EFSA) with support from the Joint Research Centre (JRC). Guidance for the identification of endocrine disruptors in the context of Regulations (EU) No 528/2012 and (EC) No 1107/2009 (Pre-publication version; June 2018)

[CR12] ECHA (2017) Risk Management Option Analysis Conclusion Document. Substance Name: Terephtalic Acid. France, January 2017. https://echa.europa.eu/documents/10162/e6767a04-5a3c-e73d-be91-4f041d2bf5d5.

[CR13] Eilstein J, Grégoire S, Fabre A (2020). Use of human liver and EpiSkin™ S9 subcellular fractions as a screening assays to compare the in vitro hepatic and dermal metabolism of 47 cosmetics-relevant chemicals. J Appl Toxicol.

[CR15] EU (2009) Regulation (EC) No 1223/2009 of the European Parliament and of the Council of 30 November 2009 on cosmetic products. http://data.europa.eu/eli/reg/2009/1223/oj.

[CR16] George JD, Price CJ, Marr MC, Myers CB, Jahnke GD (2001). Evaluation of the developmental toxicity of isoeugenol in Sprague-Dawley (CD) rats. Toxicol Sci.

[CR17] Hewitt NJ, Troutman J, Przibilla J (2022). Use of in vitro metabolism and biokinetics assays to refine predicted in vivo and in vitro internal exposure to the cosmetic ingredient, phenoxyethanol, for use in risk assessment. Regul Toxicol Pharmacol.

[CR18] Ji J-z, Lao K-j, Hu J (2014). Discovery of novel aromatase inhibitors using a homogeneous time-resolved fluorescence assay. Acta Pharmacol Sin.

[CR19] Kleinstreuer NC, Ceger P, Watt ED (2017). Development and validation of a computational model for androgen receptor activity. Chem Res Toxicol.

[CR20] Kolšek K, Mavri J, Sollner Dolenc M, Gobec S, Turk S (2014). Endocrine disruptome–an open source prediction tool for assessing endocrine disruption potential through nuclear receptor binding. J Chem Inf Model.

[CR21] Kurata Y, Tabata Y, Shinei R (2005). Endocrinological properties of two novel nonsteroidal progesterone receptor modulators, CP8816 and CP8863. J Pharmacol Exp Ther.

[CR22] Lester C, Hewitt NJ, Müller-Vieira U (2021). Metabolism and plasma protein binding of 16 straight- and branched-chain parabens in in vitro liver and skin models. Toxicol in Vitro.

[CR23] Lu WJ, Desta Z, Flockhart DA (2012). Tamoxifen metabolites as active inhibitors of aromatase in the treatment of breast cancer. Breast Cancer Res Treat.

[CR24] Lui K, Tamura T, Mori T, Zhou D, Chen S (2008). MCF-7aro/ERE, a novel cell line for rapid screening of aromatase inhibitors, ERalpha ligands and ERRalpha ligands. Biochem Pharmacol.

[CR25] Mansouri K, Abdelaziz A, Rybacka A (2016). CERAPP: collaborative estrogen receptor activity prediction project. Environ Health Perspect.

[CR26] Mansouri K, Kleinstreuer N, Abdelaziz AM (2020). CoMPARA: collaborative modeling project for androgen receptor activity. Environ Health Perspect.

[CR27] Mansouri K, Grulke C, Judson R, Williams AJ (2018) OPERA models for predicting physicochemical properties and environmental fate endpoints. J Cheminformatics 10.1186/s13321-018-0263-1 10.1186/s13321-018-0263-1 PMC5843579 29520515

[CR28] Mullur R, Liu YY, Brent GA (2014). Thyroid hormone regulation of metabolism. Physiol Rev.

[CR29] Nafie MS, Elghazawy NH, Owf SM, Arafa K, Abdel-Rahman MA, Arafa RK (2022). Control of ER-positive breast cancer by ERα expression inhibition, apoptosis induction, cell cycle arrest using semisynthetic isoeugenol derivatives. Chem Biol Interact.

[CR30] OECD (2018) Revised guidance document 150 on standardised test guidelines for evaluating chemicals for endocrine disruption

[CR31] OECD (2020) Test No. 458: stably transfected human androgen receptor transcriptional activation assay for detection of androgenic agonist and antagonist activity of chemicals

[CR32] OECD (2021) Test No. 455: performance-based test guideline for stably transfected transactivation in vitro assays to detect estrogen receptor agonists and antagonists

[CR33] OECD (2022) Test No. 456: H295R steroidogenesis assay

[CR34] Ouedraogo G, Alexander-White C, Bury D (2022). Read-across and new approach methodologies applied in a 10-step framework for cosmetics safety assessment—a case study with parabens. Regul Toxicol Pharmacol.

[CR35] Park Y, Park J, Lee HS (2021). Endocrine disrupting potential of veterinary drugs by in vitro stably transfected human androgen receptor transcriptional activation assays. Environ Pollut.

[CR36] Patisaul HB, Fenton SE, Aylor D (2018). Animal models of endocrine disruption. Best Pract Res Clin Endocrinol Metab.

[CR37] Routledge EJ, Sumpter JP (1996). Estrogenic activity of surfactants and some of their degradation products assessed using a recombinant yeast screen. Environ Toxicol Chem.

[CR38] Sohoni P, Sumpter J (1998) Several environmental oestrogens are also anti-androgens. J Endocrinol 158, 327-339. 10.1677/joe.0.1580327 10.1677/joe.0.1580327 9846162

[CR39] Sonneveld E, Jansen HJ, Riteco JA, Brouwer A, van der Burg B (2005). Development of androgen- and estrogen-responsive bioassays, members of a panel of human cell line-based highly selective steroid-responsive bioassays. Toxicol Sci.

[CR14] USEPA CompTox Chemicals Dashboard https://www.epa.gov/chemical-research/comptox-chemicals-dashboard.

[CR40] Valerio LG (2009). In silico toxicology for the pharmaceutical sciences. Toxicol Appl Pharmacol.

[CR6] van der Burg B, van der Linden S, Man H-y et al. (2013) A Panel of Quantitative Calux® Reporter Gene Assays for Reliable High-Throughput Toxicity Screening of Chemicals and Complex Mixtures High‐Throughput Screening Methods in Toxicity Testing. p 519–532

[CR41] van der Ven LT, Wester PW, Vos JG (2003). Histopathology as a tool for the evaluation of endocrine disruption in zebrafish (Danio rerio). Environ Toxicol Chem.

[CR42] van Vugt-Lussenburg BMA, van der Lee RB, Man HY (2018). Incorporation of metabolic enzymes to improve predictivity of reporter gene assay results for estrogenic and anti-androgenic activity. Reprod Toxicol.

[CR43] Verheyen GR, Braeken E, Van Deun K, Van Miert S (2017). Evaluation of in silico tools to predict the skin sensitization potential of chemicals. SAR QSAR Environ Res.

[CR44] Weyrich A, Joel M, Lewin G, Hofmann T, Frericks M (2022). Review of the state of science and evaluation of currently available in silico prediction models for reproductive and developmental toxicity: a case study on pesticides. Birth Defects Res.

[CR45] WHO (2012) State of the science of endocrine disrupting chemicals 2012.Edited by Åke Bergman, Jerrold J. Heindel, Susan Jobling, Karen A. Kidd and R. Thomas Zoeller. https://apps.who.int/iris/bitstream/handle/10665/78102/WHO_HSE_PHE_IHE_2013.1_eng.pdf.

[CR46] Yang H, Lou C, Sun L, et al. (2018) admetSAR 2.0: web-service for prediction and optimization of chemical ADMET properties. Bioinformatics 35(6):1067–1069 10.1093/bioinformatics/bty707 10.1093/bioinformatics/bty707 30165565

[CR47] Zakár T, Kaufmann G, Tóth M (1986). Assignment of anabolic-androgenic and antiandrogenic properties to some chlorine-substituted steroids on the basis of their binding characteristics to the androgen receptor of the rat seminal vesicle. Exp Clin Endocrinol.

[CR48] Zava DT, Landrum B, Horwitz KB, McGuire WL (1979). Androgen receptor assay with [3H]methyltrienolone (R1881) in the presence of progesterone receptors. Endocrinology.

