# Supplementary material for: Evaluation of new alternative methods for the identification of estrogenic, androgenic and steroidogenic effects: a comparative in vitro/in silico study
Source: Arch Toxicol. 2023 Oct 11;98(1):251–66. doi: 10.1007/s00204-023-03616-y (PMC10761396; doi:10.1007/s00204-023-03616-y)

Online Resource 2: Supplementary Figures

Evaluation of new alternative methods for the identification of estrogenic, androgenic and steroidogenic effects: a comparative *in vitro/in silico* study

Najjar A*, Wilm A, Meinhardt J, Mueller N, Boettcher M, Ebmeyer J, Schepky, A., Lange, D.*

* Contributed equally

Beiersdorf, Unnastrasse 48, 20245 Hamburg, Germany

Corresponding author:

Abdulkarim Najjar

Email: abdulkarim.najjar@beiersdorf.com

ORCID 0000-0003-4620-9830

Supplementary Figure 1A. Results of the YES ER agonist and antagonist assays. 17-β Estradiol was the positive control for agonistic effects and 4-hydroxytamoxifen was the positive control for antagonist effects (denoted by closed circles). The test chemical was tested in 2 independent assays, Assay 1 = blue circles and Assay 2 = red symbols. Values are for non-cytotoxic concentrations and are a mean of duplicate replicates performed in two independent experiments. The values for the reference chemical are a mean ± SD from 4 replicates.

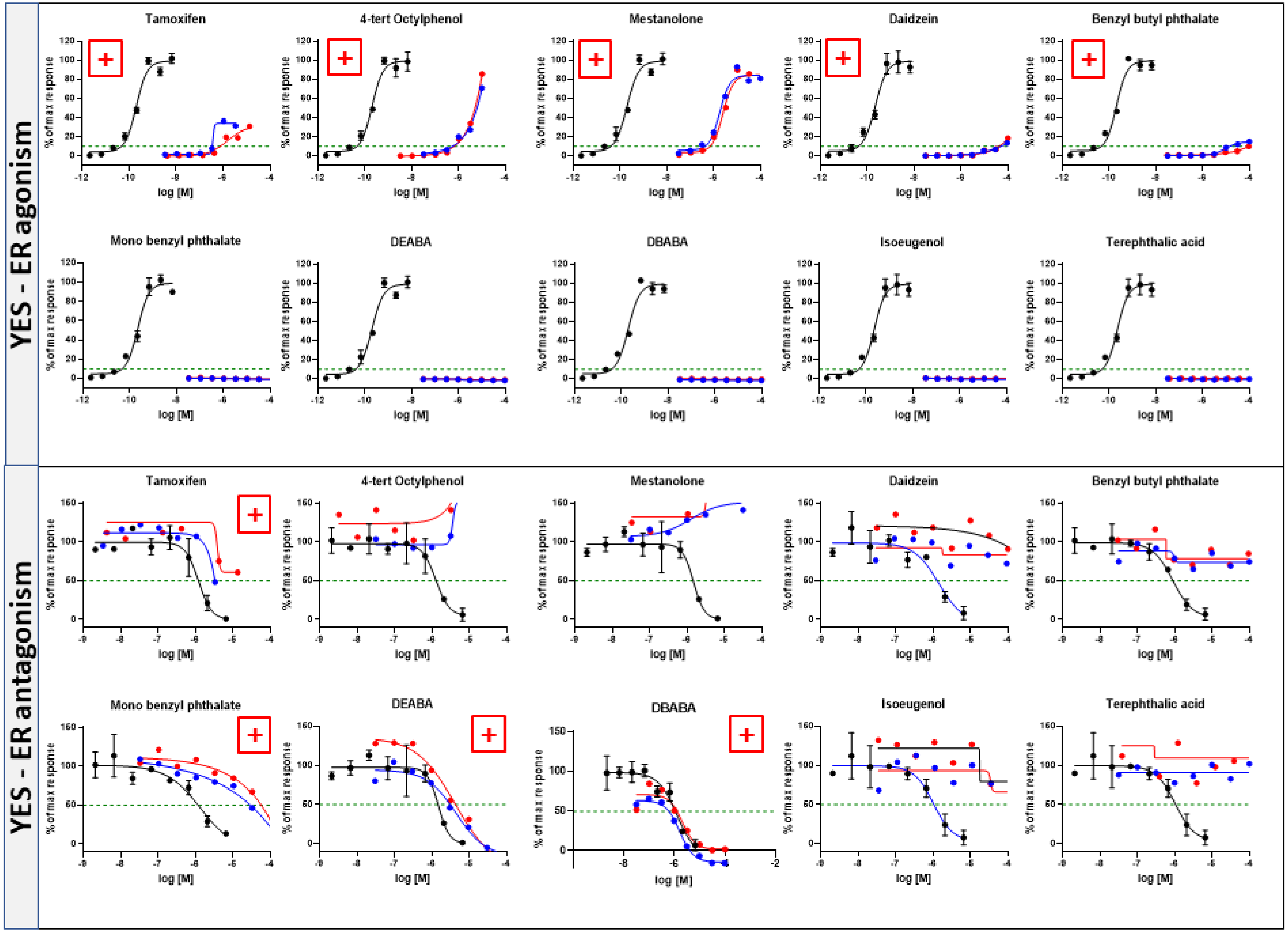

Supplementary Figure 1B. Results of the YAS AR agonist and antagonist assays. 5α-dihydrotestosterone was the positive control for agonistic effects and flutamide was the positive control for antagonist effects (denoted by closed circles). The test chemical was tested in 2 independent assays: Assay 1 = blue circles and Assay 2 = red symbols. Values are a mean of duplicate replicates performed in two independent experiments. The values for the reference chemical are a mean ± SD from 4 replicates.

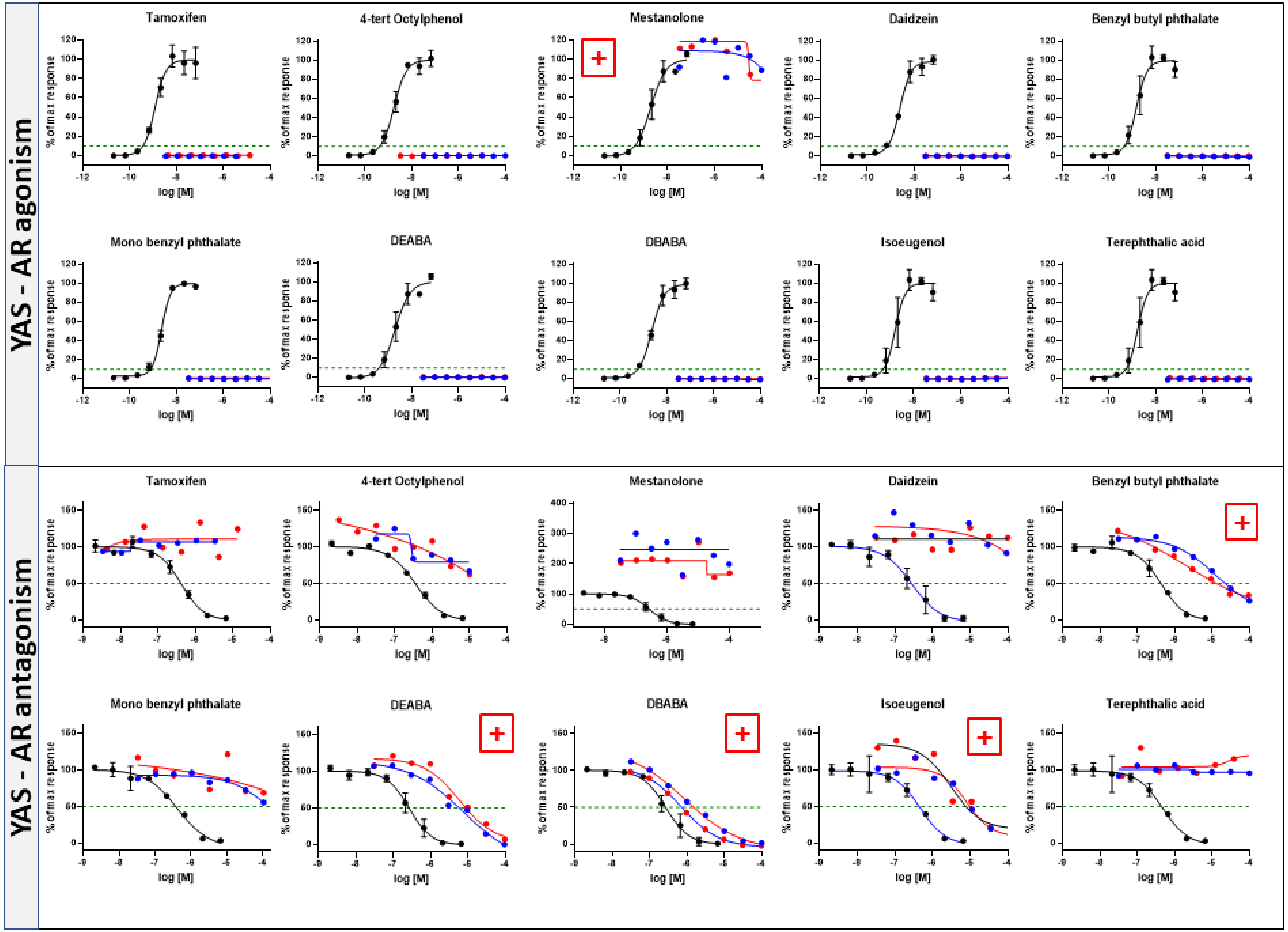

Supplementary Figure 2A. Cytotoxicity of the test chemicals and positive controls in cells used for the CALUX transactivation assays. Tributyltin acetate was the positive cytotoxicity control (denoted by closed circles). The test chemical was tested in triplicates in 2 assays, Assay 1 = blue circles and Assay 2 = red symbols. Values are a mean ± SD.

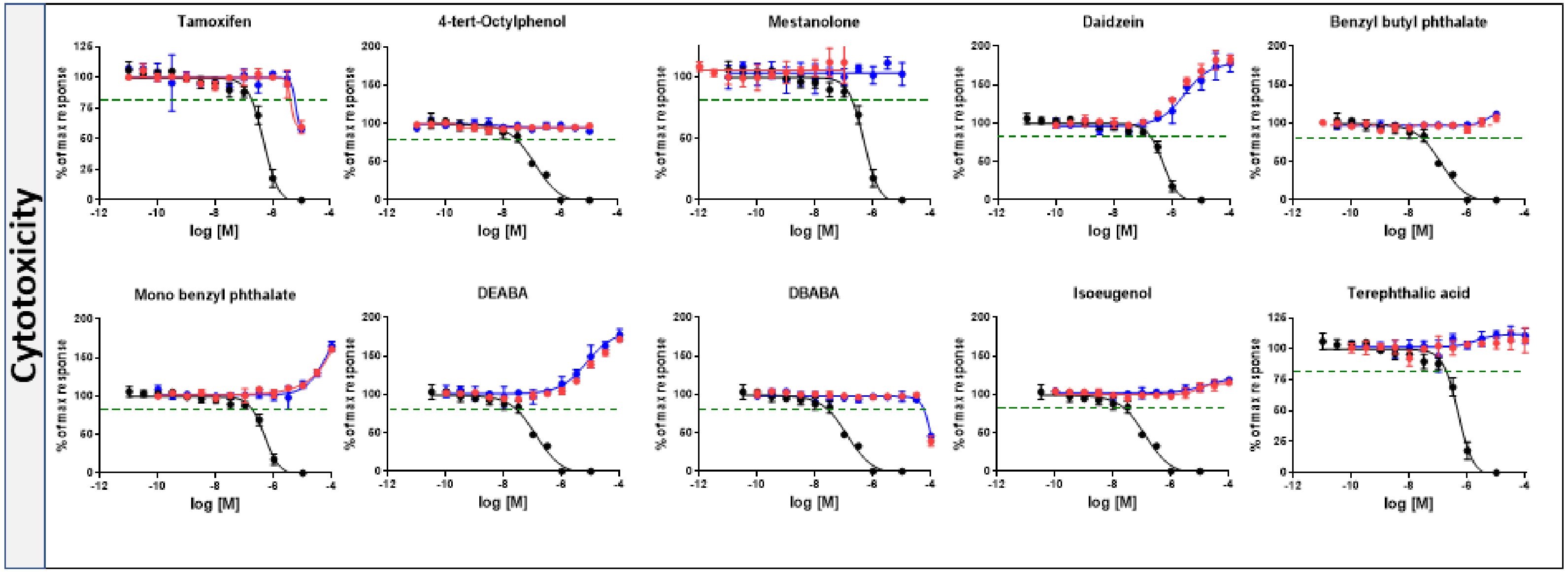

Supplementary Figure 2B Results of the CALUX transactivation ER agonist and antagonist assays. Estradiol was the positive control for agonistic effects and tamoxifen was the positive control for antagonist effects (denoted by closed circles). The test chemical was tested in 2 independent assays n=3, Assay 1 = blue circles and Assay 2 = red symbols. Values are a mean ± SD.

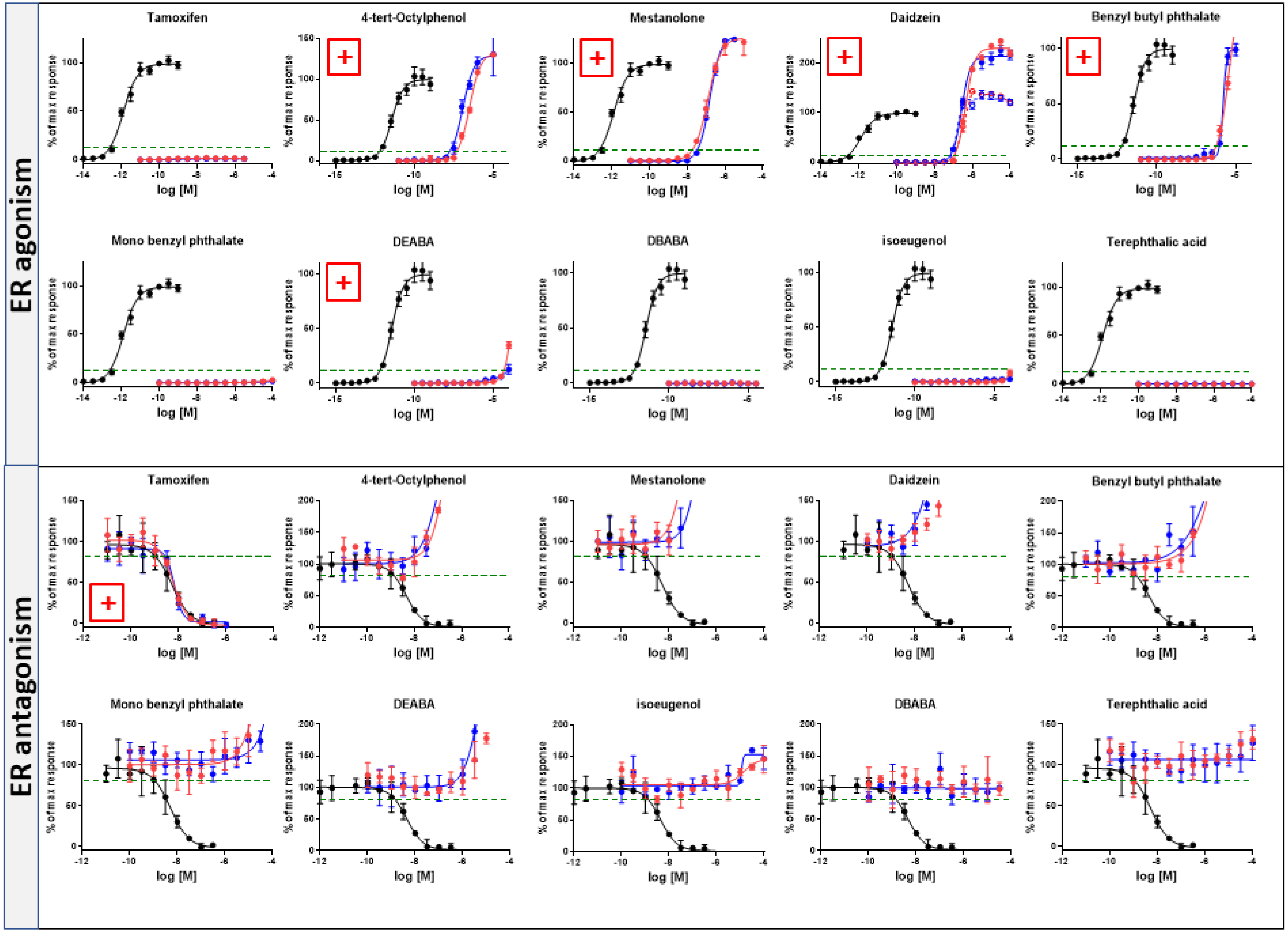

Supplementary Figure 2C Results of the CALUX transactivation AR agonist and antagonist assays. Dihydrotestosterone was the positive control for agonistic effects and flutamide was the positive control for antagonist effects (denoted by closed circles). The test chemical was tested in 2 independent assays n=3, Assay 1 = blue circles and Assay 2 = red symbols. Values are a mean ± SD.

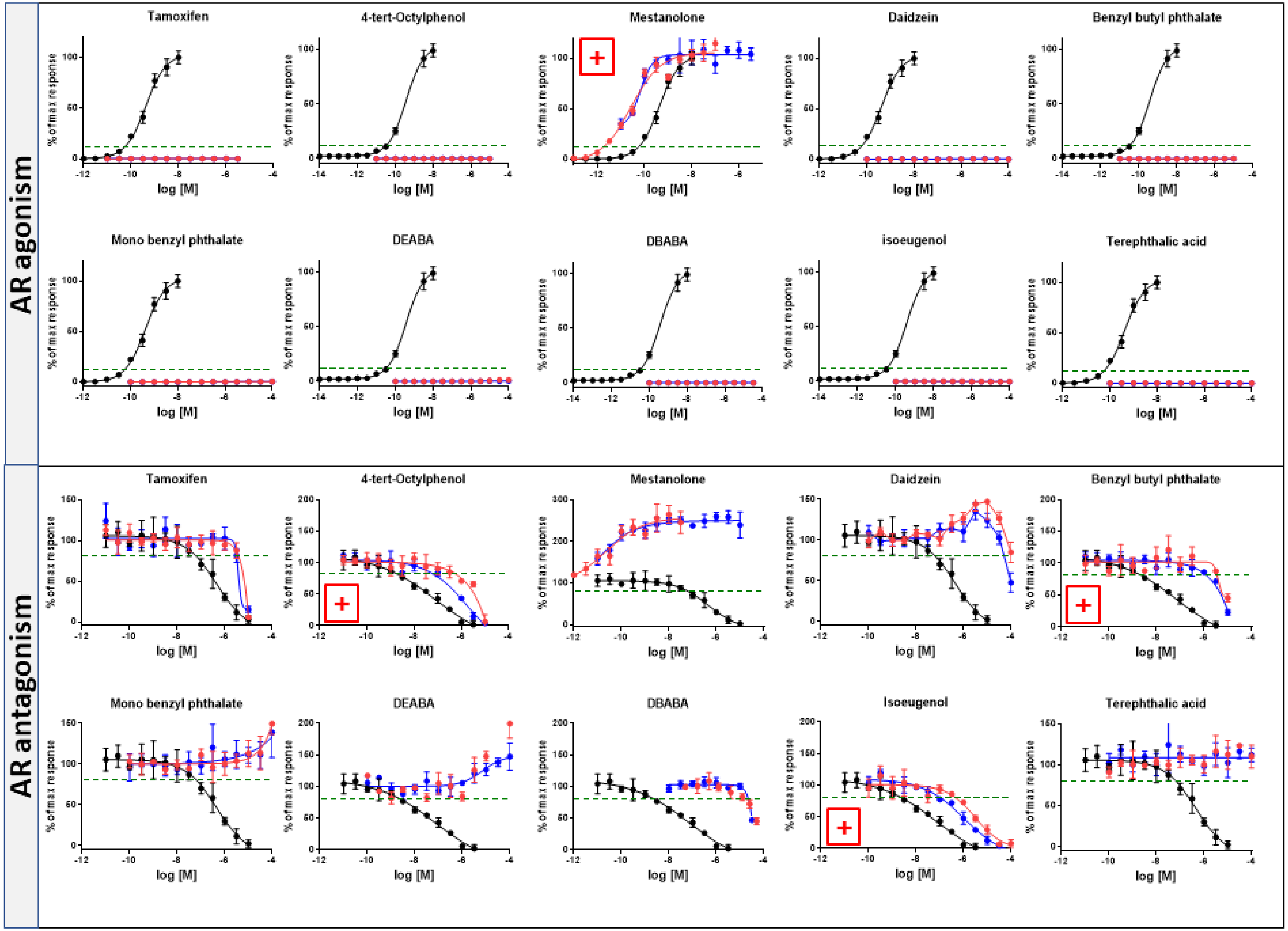

Supplementary Figure 2D Results of the H295R steroidogenesis assays for estrogenicity and androgenicity. The test chemical was tested in 2 independent assays n=3, Assay 1 = blue circles and Assay 2 = red symbols. Values are a mean ± SD.

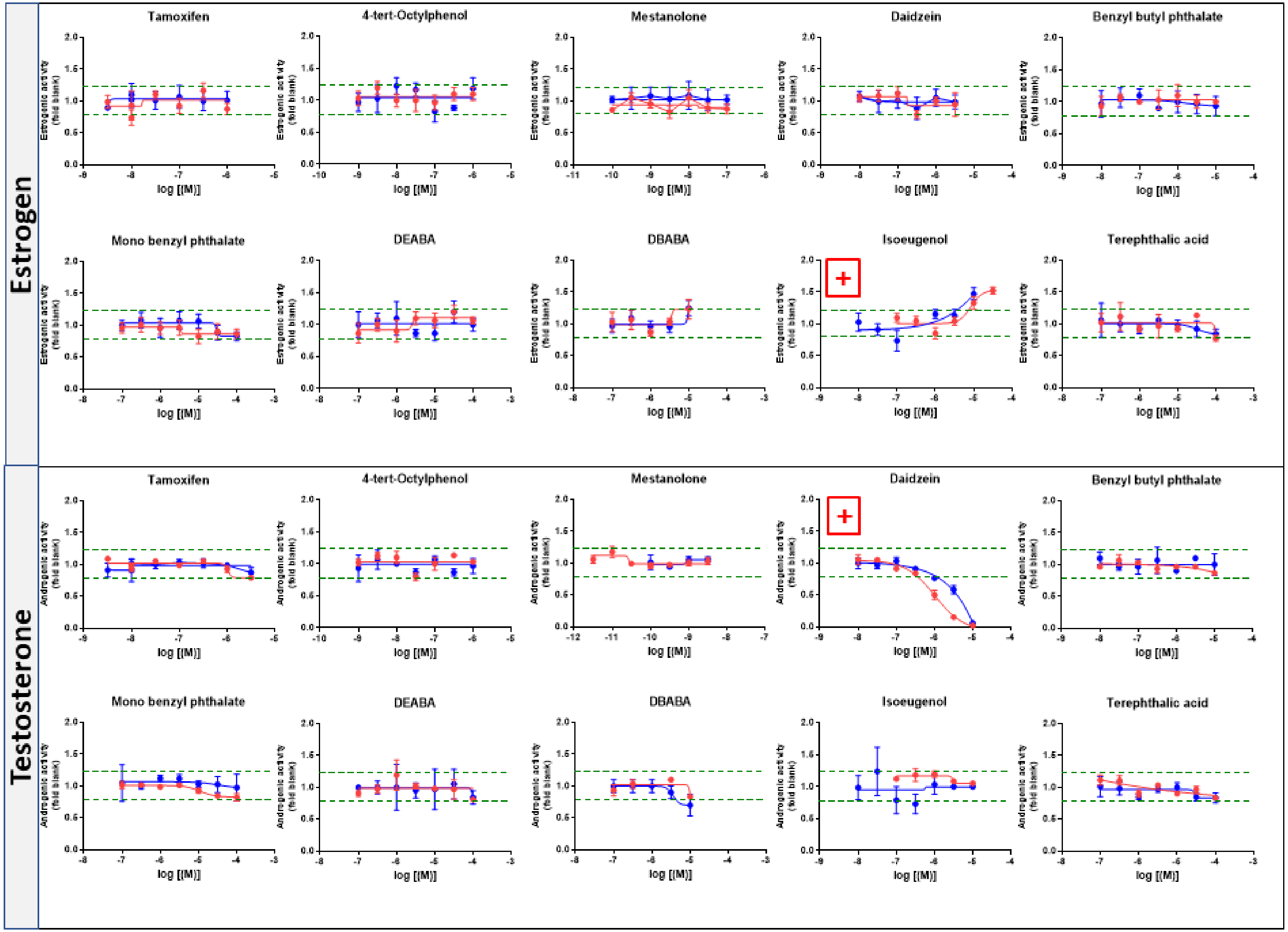

Supplement: Supplementary file 2 — Supplementary file2 (PDF 614 KB) [file 204_2023_3616_MOESM2_ESM.pdf]
