# Supplementary material for: Evaluation of new alternative methods for the identification of estrogenic, androgenic and steroidogenic effects: a comparative in vitro/in silico study
Source: Arch Toxicol. 2023 Oct 11;98(1):251–66. doi: 10.1007/s00204-023-03616-y (PMC10761396; doi:10.1007/s00204-023-03616-y)
Supplement: Supplementary file 3 — Supplementary file3 (DOCX 41 KB) [file 204_2023_3616_MOESM3_ESM.docx]

**Online Resource 3: *In vitro* methods**

**Evaluation of new alternative methods for the identification of estrogenic, androgenic and steroidogenic effects: a comparative *in vitro/in silico* study**

Najjar A*, Wilm A, Meinhardt J, Mueller N, Boettcher M, Ebmeyer J, Schepky, A., Lange, D

Beiersdorf, Unnastrasse 48, 20245 Hamburg, Germany

***Corresponding author**:

Abdulkarim Najjar

Email: abdulkarim.najjar@beiersdorf.com

ORCID 0000-0003-4620-9830

***In vitro* measurements**

**YES/YAS assays**

The YES/YAS assays are intended for screening of chemicals in a higher throughput colorimetric plate reader assay. In the YES assay, human ERα is integrated in the yeast (*Saccharomyces cerevisiae*) chromosome, and for the YAS assay human AR is integrated (Routledge and Sumpter 1996; Sohoni and Sumpter 1998). The cells have a plasmid containing lacZ coding for β-galactosidase under the control of estrogen or androgen response elements (REs); therefore, a chemical that binds to the receptor induces the synthesis of β-galactosidase. This enzyme then converts a yellow substrate to a purple metabolite, which can be measured colorimetrically.

The YES/YAS XL (Xtra Lyticase) assay kit was from Xenometrix AG (Allschwil, Switzerland) and was conducted according to the Supplier’s instructions. Growing yeast cells stably transformed with either hERα (YES) or hAR (YAS) and a β-galactosidase reporter system were exposed to a range of concentrations of each test compound. Of note, the test concentration ranges tested were same as those used in the CALUX assays. The positive control chemicals for agonistic effects were 17-β estradiol for YES and 5α-dihydrotestosterone for YAS. The positive control chemicals for antagonistic effects were 4-hydroxytamoxifen for YES and flutamide for YAS. A combination of a fixed concentration of positive control and a range of concentrations of each test chemical were tested. The cells were incubated with each treatment for 18 h at 31°C. The cells were lysed in the presence of the yellow β-galactosidase-substrate, chlorophenol red-β-D-galactopyranoside (CPRG). Yeast cell growth was assessed prior to addition of the lysis buffer by measuring the absorbance at 690 nm. The production of the red metabolite of CPRG was determined by measuring the absorbance at 570 nm. It was corrected for diffraction by cells and debris by a simultaneous measurement at 690 nm. The results were evaluated for estrogenic and androgenic agonistic and antagonistic activity, as well as for yeast growth inhibition or cytotoxicity.

$$Growth factor G= \frac{A_{690,S}}{A_{690, VC}}$$

$$Induction ratio \left( IR \right)= \frac{1}{G} \times\frac{A_{570, S}}{A_{570,VC}}$$

where A_690,S_ is the absorbance of the sample at 690 nm before lysis; A_690,VC_ is the absorbance of the vehicle control at 690 nm before lysis; A_570,S_ is the net absorbance of the sample at 570 nm – 690 nm after lysis; A_570,VC_ is the net absorbance of the vehicle control at 570 nm – 690 nm after lysis.

An IR value ≥ IR10 in the YES or YAS agonist assay indicates an agonistic effect (IR10 is defined as the IR which is 10% of (IR max - IR solvent) above the IR solvent. An IR value ≤ IR50 in the YES or YAS antagonist assay indicates a possible antagonistic effect (IR50 is defined as 50% of (IR control - IR solvent), where the IR control is at the corresponding fixed concentration of agonist used: 3.3 × 10^-10^ M 17-β estradiol and 3.3 × 10^-9^ M 5α-dihydrotestosterone, respectively).

Values shown in the figures are for non-cytotoxic concentrations and are a mean of duplicate replicates performed in two independent experiments. The values for the reference chemical are from 4 replicates.

**Receptor binding assays**

ED receptor binding assays typically involve exposing cells that express the receptors to the test chemical and measuring the ability of the chemical to replace a radiolabeled ligand, thereby assessing its binding affinity (Bowes et al. 2012).

The chemicals were tested for their ability to bind to different targets. In radioligand assays, targets included the binding affinity to human (non-selective) ER and human AR (Kurata et al. 2005; Zava et al. 1979). Chemicals were incubated at 10 µM with cells overexpressing ER or AR for 24 h at 4°C, after which, the binding of the radiolabeled ligand ([^3^H]estradiol for ER and [^3^H]methyltrienolone for AR) was measured by liquid scintillation counting. An increase or decrease higher than 50% were considered to represent significant effects. Four chemicals were tested in additional incubations at several concentrations up to the maximum soluble concentration for IC50 or EC50 determination for the associated target. Receptor binding was calculated as a % inhibition of the binding of a radioactively labeled ligand specific for each target.

Each test chemical was tested in a single assay in duplicate.

**CALUX Receptor-Transactivation-Assays**

The human CALUX® assays were used to measure the impact of test compounds on estrogen and androgen pathways. These assays are based on a direct or indirect interaction of the test chemical with a specific receptor that regulates the transcription of a reporter gene product (Sonneveld et al. 2005; Sonneveld et al. 2006; van der Burg et al. 2013). Chemicals can activate (or inactivate, in the case of an antagonistic activity) a ligand-specific pathway by binding to the receptor (ER, AR), after which the receptor-ligand complex binds to specific DNA REs as a reporter gene, resulting in increased (or decreased) cellular expression of a marker protein (e.g., luciferase). These have been used to measure a wide variety of products and, importantly, have also been validated and are already incorporated in relevant OECD test guidelines (OECD 2020; OECD 2021).

Chemicals were dissolved and diluted in DMSO. The highest stock concentration was according to the solubility in assay medium, which was evaluated by diluting the DMSO stock in CALUX assay medium (1% v/v). The cytotoxicity of each test chemical was assessed using the U2-OS based cytotoxicity CALUX bioassay. Concentrations causing >20% reduction of luminescence were considered cytotoxic. Only dilutions that did not show any signs of cytotoxicity (relative induction in the cytotoxicity CALUX bioassay > 80%) were used for final evaluation of analysis results. For the (anti-)ERα and (anti-)AR CALUX assays, CALUX cells were seeded in assay medium and then exposed to a range of concentrations of each test chemical and positive control for 24 h (triplicates). Luciferase production was quantified by measuring luminescence following addition of luciferin. For antagonist assays, a fixed concentration of receptor agonist was co-incubated with a range of concentrations of the test chemical. The assay medium was analyzed as described previously (Sonneveld et al. 2005; van der Burg et al. 2013). Estradiol was the positive control for ER agonistic effects and tamoxifen was the positive control for ER antagonist effects. For the AR assays, dihydrotestosterone was the positive control for agonistic effects and flutamide was the positive control for antagonist effects.

Each test chemical was tested in triplicates in two independent experiments.

An additional assay was conducted for BBP tested in the ER and AR CALUX assays with metabolic supplements added (according to (van Vugt-Lussenburg et al. 2018)). Incubations contained rat induced liver S9 with NADPH, and in the presence of NADPH with and without the Phase 2 cofactors, reduced glutathione, PAPS and UDPGA.

**Aromatase assay**

An aromatase inhibition assay was also included in the *in vitro* assay panel. This enzyme – otherwise known as CYP19A1 - is responsible for converting androgens into estrogens (Blakemore and Naftolin 2016; Bulun 2014); therefore, chemicals that inhibit aromatase activity can lead to reduced estrogen production, which can affect reproductive and developmental processes (Kragie 2002; Tiboni and Ponzano 2016).

Aromatase activity was measured by incubating the substrate, testosterone (20 nM), with human recombinant CYP19A1 and NADPH in the presence and absence of 10 µM test chemical for 5 min at 37°C. The metabolite, estradiol, was measured by time-resolved fluorescence according to the method described previously (Ji et al. 2014). Enzyme inhibition was calculated as a % inhibition of control enzyme activity.

Each test chemical was tested in a single assay in duplicate.

**H295R Steroidogenesis assay**

The *in vitro* H295R Steroidogenesis Assay is based on the human adenocarcinoma cell line, NCI-H295R, in which the human steroidogenesis pathway is fully functional (OECD 2022). The effect of the test chemical on the production of 17β-estradiol and testosterone is measured using ERα CALUX and AR CALUX technology.

The impact of test chemicals on steroidogenesis was measured according to estrogen and androgen production by H295R cells, according to the OECD test guideline 456 (OECD 2022). H295R cells were seeded in 48-well plates in assay medium, and treated with a range of concentrations of each test chemical in triplicate for 48 h. A sample was considered ‘active’ if it decreased the hormone production by 0.8-fold or increased the hormone production 1.2-fold.

The test chemical was tested in two independent assays, each in triplicate.

**References**

Blakemore J, Naftolin F (2016) Aromatase: Contributions to Physiology and Disease in Women and Men. Physiology (Bethesda) 31(4):258-69 doi:10.1152/physiol.00054.2015

Bowes J, Brown AJ, Hamon J, et al. (2012) Reducing safety-related drug attrition: the use of in vitro pharmacological profiling. Nature Reviews Drug Discovery 11(12):909-922 doi:10.1038/nrd3845

Bulun SE (2014) Aromatase and estrogen receptor α deficiency. Fertil Steril 101(2):323-9 doi:10.1016/j.fertnstert.2013.12.022

Ji J-z, Lao K-j, Hu J, et al. (2014) Discovery of novel aromatase inhibitors using a homogeneous time-resolved fluorescence assay. Acta Pharmacologica Sinica 35(8):1082-1092 doi:10.1038/aps.2014.53

Kragie L (2002) Aromatase in primate pregnancy: a review. Endocr Res 28(3):121-8 doi:10.1081/erc-120015041

Kurata Y, Tabata Y, Shinei R, et al. (2005) Endocrinological Properties of Two Novel Nonsteroidal Progesterone Receptor Modulators, CP8816 and CP8863. Journal of Pharmacology and Experimental Therapeutics 313(2):916-920 doi:10.1124/jpet.104.074146

OECD (2020) Test No. 458: Stably Transfected Human Androgen Receptor Transcriptional Activation Assay for Detection of Androgenic Agonist and Antagonist Activity of Chemicals,

OECD (2021) Test No. 455: Performance-Based Test Guideline for Stably Transfected Transactivation In Vitro Assays to Detect Estrogen Receptor Agonists and Antagonists,

OECD (2022) Test No. 456: H295R Steroidogenesis Assay,

Routledge EJ, Sumpter JP (1996) Estrogenic activity of surfactants and some of their degradation products assessed using a recombinant yeast screen. Environmental Toxicology and Chemistry 15(3):241-248 doi:<https://doi.org/10.1002/etc.5620150303>

Sohoni P, Sumpter J (1998) Sohoni, P. & Sumpter, J.P. Several environmental oestrogens are also anti-androgens. J. Endocrinol. 158, 327-339. The Journal of endocrinology 158:327-39 doi:10.1677/joe.0.1580327

Sonneveld E, Jansen HJ, Riteco JA, Brouwer A, van der Burg B (2005) Development of androgen- and estrogen-responsive bioassays, members of a panel of human cell line-based highly selective steroid-responsive bioassays. Toxicol Sci 83(1):136-48 doi:10.1093/toxsci/kfi005

Sonneveld E, Riteco JA, Jansen HJ, et al. (2006) Comparison of in vitro and in vivo screening models for androgenic and estrogenic activities. Toxicol Sci 89(1):173-87 doi:10.1093/toxsci/kfj009

Tiboni GM, Ponzano A (2016) Fetal safety profile of aromatase inhibitors: Animal data. Reprod Toxicol 66:84-92 doi:10.1016/j.reprotox.2016.09.016

van der Burg B, van der Linden S, Man H-y, et al. (2013) A Panel of Quantitative Calux® Reporter Gene Assays for Reliable High-Throughput Toxicity Screening of Chemicals and Complex Mixtures High‐Throughput Screening Methods in Toxicity Testing. p 519-532

van Vugt-Lussenburg BMA, van der Lee RB, Man HY, et al. (2018) Incorporation of metabolic enzymes to improve predictivity of reporter gene assay results for estrogenic and anti-androgenic activity. Reprod Toxicol 75:40-48 doi:10.1016/j.reprotox.2017.11.005

Zava DT, Landrum B, Horwitz KB, McGuire WL (1979) Androgen receptor assay with [3H]methyltrienolone (R1881) in the presence of progesterone receptors. Endocrinology 104(4):1007-12 doi:10.1210/endo-104-4-1007
